# Homoeolog expression divergence contributes to time of day changes in transcriptomic and glucosinolate responses to prolonged water limitation in *Brassica napus*

**DOI:** 10.1111/tpj.70011

**Published:** 2025-02-24

**Authors:** Angela Ricono, Ella Ludwig, Anna L. Casto, Stevan Zorich, Joshua Sumner, Kevin Bird, Patrick P. Edger, Todd C. Mockler, Adrian D. Hegeman, Malia A. Gehan, Kathleen Greenham

**Affiliations:** ^1^ University of Minnesota St. Paul Minnesota 55108 USA; ^2^ Donald Danforth Plant Science Center St. Louis Missouri 63132 USA; ^3^ Michigan State University East Lansing Michigan 48824 USA

**Keywords:** water limitation, *Brassica napus*, diel, high‐throughput phenotyping, transcriptomics, glucosinolates, homoeolog

## Abstract

Water availability is a major determinant of crop production, and rising temperatures from climate change are leading to more extreme droughts. To combat the effects of climate change on crop yields, we need to develop varieties that are more tolerant to water‐limited conditions. We aimed to determine how diverse crop types (winter/spring oilseed, tuberous, and leafy) of the allopolyploid *Brassica napus*, a species that contains the economically important rapeseed oilseed crop, respond to prolonged water limitation. We exposed plants to an 80% reduction in water and assessed growth and color on a high‐throughput phenotyping system over 4 weeks and ended the experiment with tissue collection for a time course transcriptomic study. We found an overall reduction in growth across cultivars but to varying degrees. Diel transcriptome analyses revealed significant accession‐specific changes in time‐of‐day regulation of photosynthesis, carbohydrate metabolism, and sulfur metabolism. Interestingly, there was extensive variation in which homoeologs from the two parental subgenomes responded to water limitation across crop types that could be due to differences in regulatory regions in these allopolyploid lines. Follow‐up experiments on select cultivars confirmed that plants maintained photosynthetic health during the prolonged water limitation while slowing growth. In two cultivars examined, we found significant time of day changes in levels of glucosinolates, sulfur‐ and nitrogen ‐rich specialized metabolites, consistent with the diel transcriptomic responses. These results suggest that these lines are adjusting their sulfur and nitrogen stores under water‐limited conditions through distinct time of day regulation.

## Significance Statement

This study explores the diversity in transcriptomic responses among 16 *Brassica napus* allopolyploid accessions subjected to a 4‐week prolonged water limitation experiment using a high‐throughput phenotyping system. We uncovered unique time of day variation in transcriptome responses across accessions that span a range of biological processes including photosynthesis, carbohydrate metabolism, and sulfur metabolism, with two oilseed accessions with altered growth under water limitation exhibiting specific diel patterns of indolic glucosinolates.

## INTRODUCTION

Climate change is reshaping daily and seasonal environmental trends causing plants to experience developmental triggers that misalign with the optimal photoperiod (Jabbur & Johnson, 2021; Oravec & Greenham, 2022). Recent examples include the early flowering of cherry blossoms in Japan (Primack et al., 2009) and early ripening of grapevines in wine‐growing regions (Gutiérrez‐Gamboa et al., 2021) both triggered by early spring warming. In addition to temperature changes, water availability continues to be a limiting factor for crops leading to a reduction in yield (Ayyaz et al., 2021; Batool et al., 2022). In 2017, it is estimated that over 58 million acres of cropland were irrigated (USDA, 2017 Census of Agriculture) thus making these crops vulnerable to crop loss. According to the UC Davis Center for Watershed Science, the 2015 drought in California accounted for $1.84 billion lost in direct costs (Howitt et al., 2014). Temperature and water availability are predicted to continue to limit the yield potential of our crops (Hasegawa et al., 2022), challenging us to develop more resistant varieties to reduce the amount of irrigated water needed.

Plants must balance growth and tolerance to overcome stress (Zhang et al., 2020). Some plants maintain or speed up growth to try and “escape” stress, while others slow growth to potentially survive beyond stress (avoidance strategy; Kooyers, 2015). This relationship is evident at the transcriptional level where the GROWTH‐REGULATING FACTOR7 (GRF7) transcription factor (TF) represses the cold and drought‐responsive DREB2A TF to inhibit stress response under normal conditions to maintain growth (Kim et al., 2012). Overexpression of DREB1A increases stress tolerance but comes at a cost of a reduction in growth and overall yield (Kasuga et al., 2004). However, when expressing DREB1A under a promoter with a time of day‐specific expression pattern, a temporal increase in expression provides stress tolerance while growth is maintained (Kasuga et al., 2004). Diel and circadian regulation of physiological and metabolic processes is an important component of abiotic stress response. The circadian gating of abiotic stress leads to time of day‐specific transcriptional responses to stress (Dong et al., 2011; Fowler et al., 2005; Graham et al., 2023). Limiting responses to certain times of day is thought to be critical for maximizing energy efficiency to maintain growth while responding to stress (Greenham & McClung, 2015). Several studies in diverse plant species have uncovered time of day‐specific responses to abiotic stress, and revealed that the largest variation in transcriptome responses is the time of day (Greenham et al., 2017; Robertson et al., 2022; Wilkins et al., 2010). Uncovering these time of day responses is critical for identifying transcriptional regulators controlling the physiological or metabolic responses associated with tolerance, yet most abiotic stress studies are done with single time point resolution. Additionally, there are limited time course studies in more complex polyploid crops where we have less understanding of the diel regulation of the transcriptome in response to stress and the amount of intraspecific variation.

*Brassica napus* (*B. napus*), a relative of Arabidopsis within Brasssicaeae, is an allopolyploid derived from the hybridization between *Brassica rapa* (*B. rapa*) and *Brassica oleracea* (*B. oleracea*) just ~7500–12 000 years ago (Chalhoub et al., 2014). The Brassica genus underwent an ancient *Brassica* whole‐genome triplication event that occurred in the ancestor of the tribe Brassicaceae, resulting in three copies of each chromosome (Lagercrantz & Lydiate, 1996). This event significantly contributed to the diversification and evolution of *Brassica* species, including important crops like cabbage, broccoli, and Brussel sprouts (Cheng et al., 2014). When two distinct species hybridize, their genetic material merges, resulting in a combined genome. The genes that were once orthologous in their separate lineages, having evolved from a common ancestral gene, are now present together within this unified genome. Due to their unique origin and relationship, these co‐existing genes are referred to as homoeologs. In this study, our analyses will focus on comparing the homoeologs between the two parental species of *B. napus*.

*B. napus* is commonly known as rapeseed and includes the second‐highest oil producing crop grown worldwide after soybean (Borges et al., 2023). In addition to spring and winter oilseed varieties, *B. napus* contains other specialty crop varieties including rutabaga and swedish turnip. Like other Brassicacea, *B. napus* produces glucosinolates (GSLs), sulfur and nitrogen‐rich specialized metabolites that are derived from amino acids such as methionine (“Aliphatics”), tryptophan (“Indolics”), or phenylalanine (“Aromatics”). These compounds are especially known for their production of the “mustard bomb,” or hydrolyzed by‐products, following herbivory (Barco & Clay, 2019). GSLs play an important role in abiotic stress responses as well. In Arabidopsis, three auxin‐sensitive Aux/IAA transcriptional repressor genes (IAA5, 6, and 19) are induced by DREBs to maintain levels of aliphatic GSLs when grown in drought conditions (Salehin et al., 2019). Under the same drought conditions, the *iaa5iaa6iaa19* mutant line exhibited reduced plant biomass but exogenous application of a single GSL (4MSO) rescued growth (Salehin et al., 2019). Sinigrin, an aliphatic GSL found in several Brassicaceae, has been shown to induce stomatal closure indicating a direct role in stomatal regulation (Zhu & Assmann, 2017). Under warming (25°C) and heat stress (32°C), Arabidopsis lines with altered GSL content, specifically lower indolic GSLs, exhibited reduced thermotolerance and lower auxin content following increased temperatures (Ludwig‐Müller et al., 2000). These results indicate an important link between GSLs and thermotolerance, auxin signaling, stomatal regulation, and drought response. Turnover of GSLs also contributes to the replenishment of primary sulfur pools (Sugiyama et al., 2021) stress responses through glutathione‐mediated reactive oxygen species (ROS) and electrophile scavenging (Zagorchev et al., 2013). Increased ROS levels will decrease photosynthetic rates thereby limiting available energy for growth (Terry, 1976). Sulfur is also a critical component in chloroplasts, primarily in the form of sulfoquinovosyl diacylglycerols (SQDGs) which are localized in the thylakoid membrane, and Fe–S proteins that aid in photosynthesis and nitrogen assimilation (Imsande, 1998). Because of the interplay between sulfur and nitrogen metabolism, the ratio between sulfur and nitrogen pools is highly regulated to maintain a strict balance. For example, a study in *B. napus* showed that when a 1:4.5 or 1:5.75 ratio of Nitrogen:Sulfur was applied, seed yield increased by 42–267%, respectively (Jamal et al., 2010; McGrath & Zhao, 1996). However, when the same amount of nitrogen was applied without sulfur, there was no increase in seed yield or seed oil, and a reduction in yield when a lower rate of nitrogen was used without sulfur (McGrath & Zhao, 1996). When soils are sulfur‐deficient, there is less nitrogen available for uptake which can promote nitrogen leaching (Jamal et al., 2010; Maršić et al., 2021). With up to 30% of sulfur allocated to GSLs in above‐ground tissues (Aghajanzadeh et al., 2014), and at least one molecule of nitrogen for each GSL, a more complete understanding of their regulation under stress conditions is critical for optimizing nutrient use efficiency in *B. napus* and other Brassicaceae crops.

Improved drought tolerance is a key target trait for the rapeseed industry (Batool et al., 2022; Khanzada et al., 2020; Raza et al., 2017) as number of siliques, number of seeds, and seed oil content are all negatively affected under water limitation (Jan et al., 2017) in addition to lower photosynthetic rates and lower vegetative biomass (Batool et al., 2022; Raza et al., 2017). In the 2023 growing season, Canada—one of the largest global producers of rapeseed—experienced its fourth year of drought with 76% of agricultural land classified as either abnormally dry (D0; water‐limited), moderately dry (D1) or extremely dry (Stephenson, 2023). In addition to temperate climates *B. napus* is grown in semi‐arid and arid zones leading to diverse ecotypes with varying responses to water limitation (Khanzada et al., 2020).

To explore how the expanded genome in *B. napus* responds to water limitation and what transcriptome level regulation drives changes in growth and metabolic processes, we performed a prolonged water limitation experiment across 16 diverse accessions of *B. napus* covering winter/summer oilseed, tuberous, and leafy crop types. In this experiment, we collected image data across the 16 diverse accessions under well‐watered (WW) and water‐limited (WL) conditions. At the end of the experiment, leaf tissue was collected at multiple time points for a diel transcriptome analysis to determine what changes to the time of day regulation of biological processes were altered under water limitation. We found that all accessions reduced their growth to various degrees but estimate that they remain photosynthetically healthy. We found unique changes in transcriptome responses across accessions that were time of day‐dependent. Most striking was the extensive variation in which homoeolog(s) encoded on the two parental subgenomes were most responsive across accessions indicating genome‐wide changes to regulatory regions in this relatively young species. In particular, two accessions had opposite regulation of GSLs suggesting unique temporal regulation of sulfur use under water limitation. A follow‐up study revealed that in fact, these accessions altered their GSL stores under water limitation resulting in different time of day changes in GSL pools.

## RESULTS

### Plants reduce growth but have higher “greenness” in non‐senescing tissue under water limitation

To identify *B. napus* accessions with improved responses to water limitation, we examined the phenotypic effects of prolonged water limitation on 16 *B. napus* accessions using the Bellwether Phenotyping Facility (Fahlgren et al., 2015). We exposed all accessions to an 80% reduction in water (20% full capacity) over 4 weeks and assessed growth and color through a high‐throughput automated system (Experimental Procedures). We used the open‐source PlantCV software on 59 850 RGB and 59 848 NIR images to quantify treatment responses.

**Figure 1:**
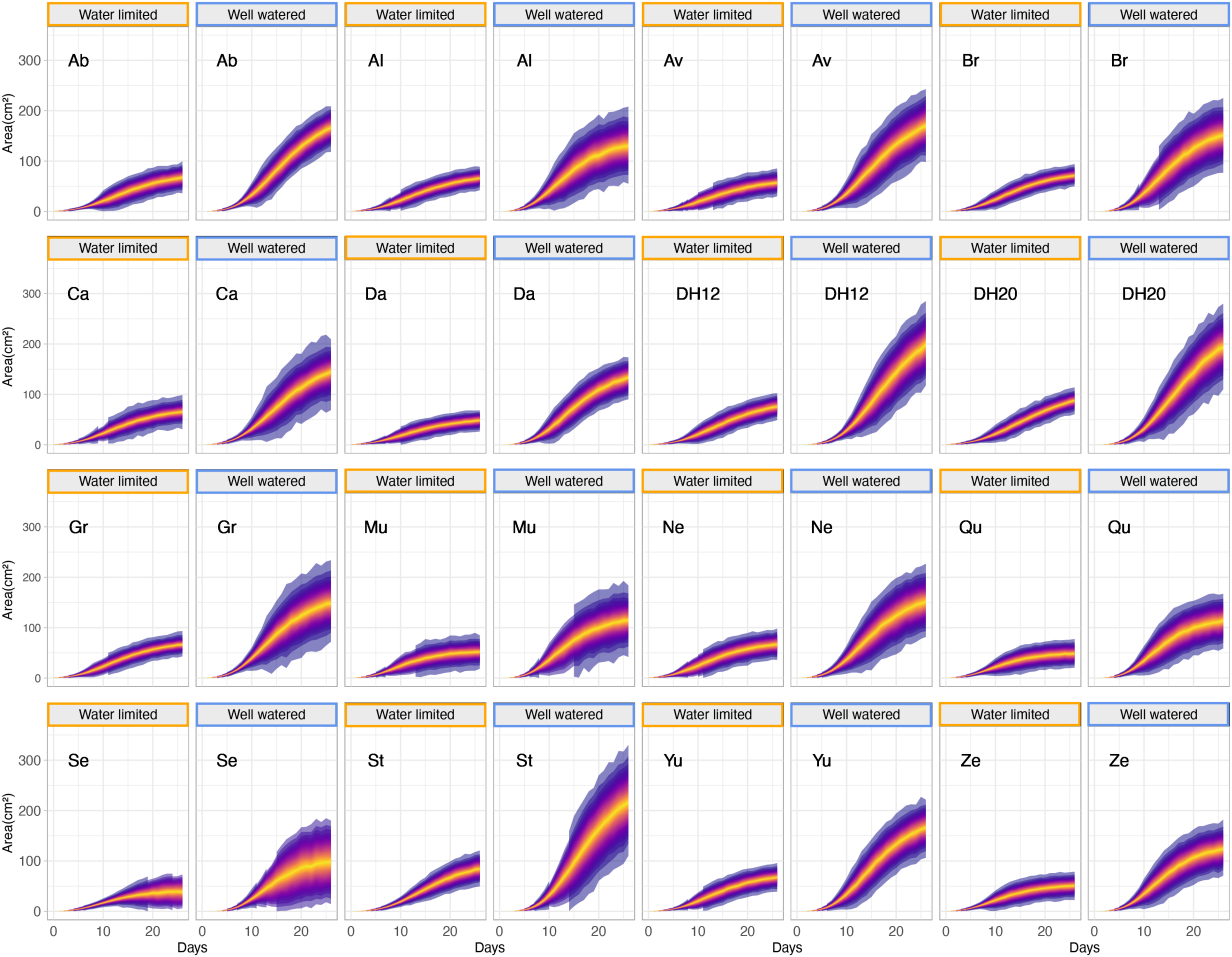
All accessions reduced plant area under 4 weeks of water limitation. Growth curves for well‐watered and water‐limited groups of each accession with Bayesian posterior predictive intervals. Bayesian hierarchical growth modeling was used to fit plant area data to a Gompertz growth model. Posterior predictive intervals are visualized for each accession and treatment (orange border, water‐limited; blue border, well‐watered). The ribbon color gradient represents the probability that the population mean falls within that interval, with the yellow area having the highest probability. Growth models of all accessions showed reduced final size in water‐limited plants. The asymptote parameter (A) was at least 50% larger in the well‐watered group for all accessions, suggesting that water limitation reduced final plant size in all accessions.

Overall, prolonged water limitation reduced the final size of all accessions (Figure 1). However, the difference in size between treatments varied among accessions with large (ex: Av; St) and small (ex: Mu; Qu) differences observed. We modeled the change in area over time (growth) using Bayesian Hierarchical models. Growth curves of all accessions in WW and WL treatments were fit to a Gompertz model (see Experimental Procedures for the equation). Using these models, we tested the hypothesis that within each accession the WW group would have a final size, represented by the Gompertz model parameter *A* (asymptote), at least 50% larger than that of the WL group. This hypothesis was supported for each accession (posterior probabilities and effect sizes reported in Table S1). The hypothesis that the inflection point in the growth curve (*B* parameter) was at least 5% larger in the WW group than in the WL group was also supported for each accession (Table S1). Lastly, the hypothesis that the growth rate (*C* parameter) of the WW group was 5% larger than the WL group was not significant for any accession (Table S1). These results show that despite the growth rate of WW and WL plants being similar, the WW plants grew larger by the end of the experiment, suggesting that water‐limitation treatment restricts growth as opposed to slowing growth rate.

While we did not directly measure photosynthesis in this initial experiment we did quantify plant color. First, we used the multiclass Naive Bayes classifier in PlantCV (Gehan et al., 2017) to categorize plant pixels into ‘healthy’ (green and purple plant pixels) and ‘senescing’ (yellow plant pixels) categories (Figure S1). We found that the percent of yellow tissue is similar or higher in the WW treatment (Figure S1). Accessions with a higher estimate of senescing tissue along with a larger overall area under WW treatment (Figure 1), likely suggest that plants are further along in development in comparison to WL plants with restricted growth.

**Figure 2:**
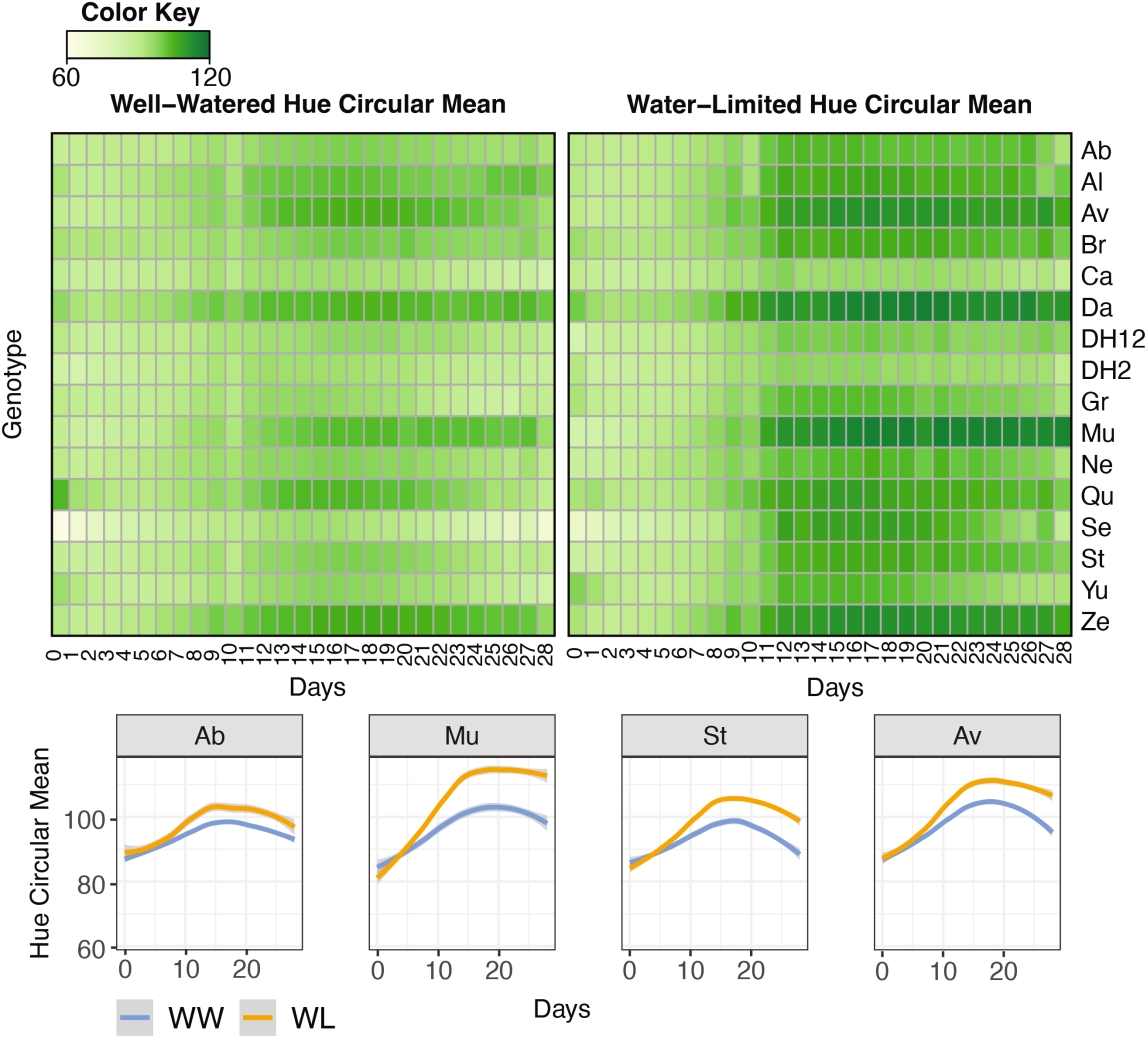
Variation in hue circular mean across accessions under water limitation. Heatmaps of average hue circular mean values of plant pixels classified as green (Top). Examples of difference in hue circular mean under water‐limited and well‐watered conditions varies across *B. napus* accessions (bottom).

We then examined the “healthy” pixels. For only the pixels categorized as “healthy” we extracted the hue circular mean. For reference, a hue value of 60° is yellow and 120° is green. Unexpectedly, we found that most WL plants were more green (values toward 120°) under prolonged water limitation in comparison to WW (Figure 2) although the magnitude of the difference varied among accessions, with relatively extreme (Mu, St, Av) and mild (Ab) differences observed. In addition, the timing of when treatments began to separate for color among the accessions differed (Figure 2). For plant growth, all lines began to deviate around day 11; however, differences in plant color were more varied. For example, Ab does not appear more green until 8 days of water limitation while Mu occurs around day 4 (Figure 2).

### NIR values are consistent with treatments

The NIR imaging system measures wavelengths of light absorbed by water. In general, lower NIR signal values suggest more water absorption because there is less signal reflected back to the NIR detector (Fahlgren et al., 2015). However, NIR signal can change with leaf thickness and tissue composition (Fahlgren et al., 2015). Our NIR imaging results match with treatment conditions (Figure S2). In nearly all accessions, the average NIR signal is lower in the WW treatment compared with the WL treatment, suggesting higher water content in WW treatment plants. Differences in NIR signal between treatments typically matched differences in plant size. For example, Mu showed smaller differences in plant size between treatments and smaller differences in NIR signal. However, accessions like Av and St had larger differences in plant size and NIR signal between treatments (Table S2). Overall, results from plant size, growth, and color suggest that *B. napus* adjusted to prolonged water limitation by slowing above‐ground growth. The reduction in growth was expected but we were surprised to find that WL plants were more green. This led us to wonder what transcriptomic changes had occurred in these lines following prolonged WL.

### Accessions exhibit variation in subgenome responsiveness that is time of day dependent

Gene expression is a dynamic process that is often under circadian or diel regulation, resulting in time of day control of physiology, metabolism, and abiotic stress response (de Barros Dantas et al., 2023; Greenham et al., 2017; Robertson et al., 2022). To capture these transcriptome dynamics, we collected leaf tissue for all accessions every 6 h over a 24 h period for RNA sequencing after 4 weeks of prolonged WL.

We first examined whether the differences in growth response under WL could be explained by the genetic background of the accessions. We called single nucleotide polymorphisms (SNPs) and clustered the accessions by PCA. Consistent with our documentation of these accessions, they grouped by crop type (Figure S3). When we mapped the difference in plant area to the PCA, we found that there was no relationship between crop type and growth response to WL conditions, indicating that the variation in growth is not explained simply by crop type. To identify significantly differentially patterned genes within each accession, we used the DiPALM R package (Greenham et al., 2020), resulting in thousands of genes across accessions with differential patterns under WL (Table S3; ‘Metadata’ tab).

**Figure 3:**
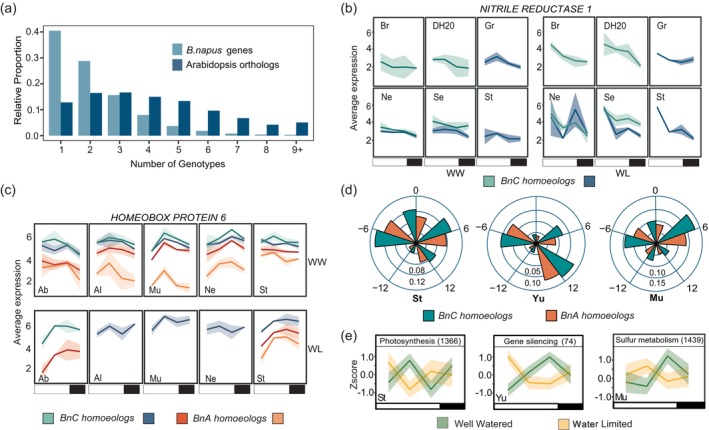
Extensive divergence in the responsive homoeolog of conserved and sugar‐responsive genes. (a) Overlap of all shared WL *B. napus* genes (light blue bars) compared with the overlap with the Arabidopsis orthologs (dark blue bars) across accessions. (b) Expression of the *NITRITE REDUCTASE 1* ortholog that was WL‐responsive in four accessions (from Panel a) with extensive variation among accessions for which homoeolog was responsive. (c) Average expression for orthologs of *HOMEOBOX PROTEIN 6* that exhibit differences in the number of WL‐responsive paralogs across accessions. (d) Distribution of phase change groups for all WL‐responsive genes by subgenome in St (large difference in growth), Yu (moderate difference in growth), and Mu (small difference in growth). Numbers outside the radial plots indicate the phase group (difference in the timing of peak expression in hours between WW and WL) and numbers inside the circles represent the relative proportion of WL genes in a phase change group relative to all WL‐responsive genes for each accession; proportions are associated with the ring directly above the number. (e) Enriched Gene Ontology process for the DiPALM clusters representing the phase groups −6 for St, +12 for Yu and +6 for Mu as shown in Panel d. Yellow lines indicate WL patterns and green are WW patterns. Shading is one standard deviation of the Zscore of gene expression. Numbers inside the parentheses indicate the number of genes in that cluster.

A comparison of the overlap of WL‐responsive genes across the 14 accessions revealed that the majority of WL‐responsive genes (~70%) are unique to one to two accessions (Figure 3a; light blue bars). However, an overlap with the Arabidopsis orthologs resulted in a higher proportion of shared genes (Figure 3a; dark blue bars) suggesting similar functional responses but variation in the homoeolog copy (encoded on either the BnA or BnC subgenome) that is responsive across accessions. We first looked into WL genes that were shared among 6 accessions since this was the relative halfway point. Within this group, we found that the *NITRITE REDUCTASE 1* ortholog is responsive to water limitation in six accessions that differ in the respective BnC paralog (Figure 3b). We compared the averaged expression levels across time points for all the genes with significantly different expression patterns under WL and found that the BnC subgenome (*B. oleracea*) had slightly higher expression than the BnA subgenome (*B. rapa*) across accessions (Figure S4). To further examine the variation in which homoeolog is responding to WL conditions, we focused on a set of previously characterized sugar‐responsive genes (Hummel et al., 2010). Overall, the majority of accessions varied in the number of genes that were responsive under WL (Table S3; Metadata tab). Four orthologs of the *HOMEOBOX PROTEIN 6* gene are expressed under WW conditions across five accessions (Ab, Al, Mu, Ne, and St). While only a single BnC homoeolog responds to WL in Al, Mu, and Ne, two respond in Ab and three respond in St (Figure 3c).

A recent study in wheat observed time of day variation in subgenome contribution to expression level under circadian conditions (Rees et al., 2022). To explore this in our dataset, we identified the time of peak expression (phase) for each gene by treatment and accession and calculated the change in phase between WW and WL. This resulted in five ‘phase groups’ that contained genes with an earlier phase under WL (negative), a later phase (positive), or no change in phase (0). Overall, the BnC subgenome contributes more for most phase change groups, but the proportion relative to the BnA subgenome varies across accessions (Figure S5). This analysis also revealed accession level differences in time of day changes of the WL transcriptome as shown by the direction of phase change under WL conditions (Figure 3d; Figure S5). For example, both St (large difference in WL growth; Figure 1) and Mu (little difference in WL growth) have the greatest change in the +6 h phase groups with overall 54 and 57% of WL‐responsive genes falling into these two groups, respectively. Yu (moderate difference in WL growth) is quite different having the largest proportion (36% of WL genes) in the +12 h group alone while other accessions had, on average, ~16% in this group. As a whole we did not find evidence that one subgenome dominates a single phase change group or growth response group, regardless of BnC having higher average expression overall.

Such large proportions of genes exhibiting similar phase changes (Figure S5) suggest coordinated regulation, and thus we would expect to find enrichment for biological pathways within a given phase change group. To explore this, we ran GO enrichment on the WL clusters from DiPALM and identified clusters that had a matching 6 h advance (St), 6 h lag (Mu), or a 12 h advance (Yu). Within these clusters (Figure 3e) we found enrichment for several biological processes including photosynthesis (St; large growth difference), sulfur metabolism (Mu; small growth difference), and gene silencing (Yu; moderate growth difference). See Table S3 ‘DiPALMClusters_GO_Terms’ tab for a full list of enriched terms. We do not find evidence for subgenome bias within enriched biological processes suggesting that both the BnA and BnC subgenomes are contributing to these pathway‐level responses.

**Figure 4:**
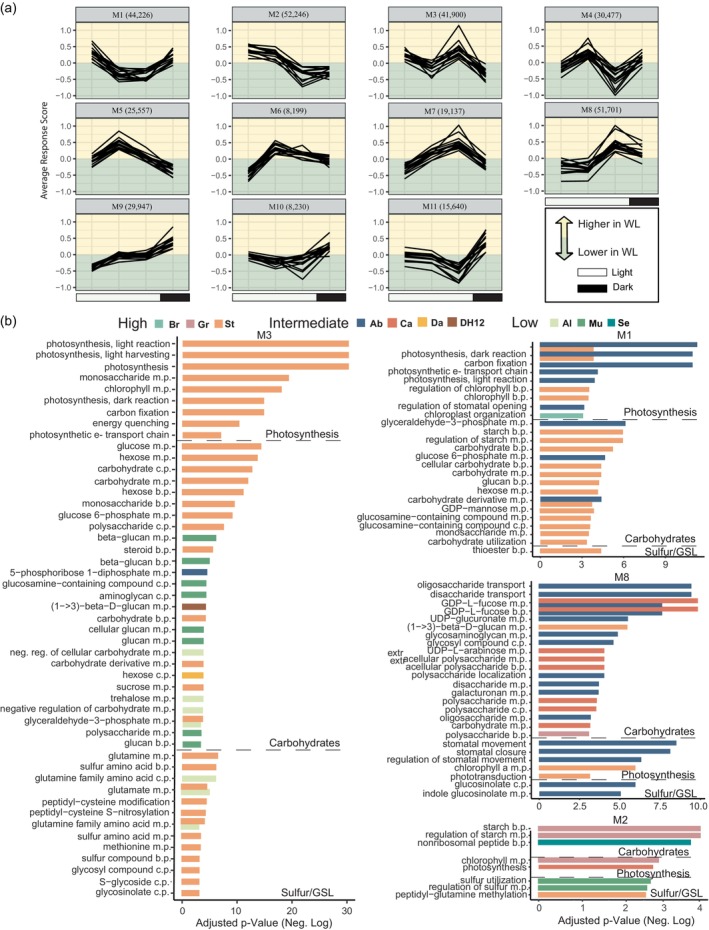
Accessions exhibit similar diel transcriptomic responses under WL but the genes underlying these patterns are enriched in different biological processes. (a) Global temporal responses to WL were determined by calculating a response score and used to build a global co‐expression network. Representative patterns (eigengenes) display variation in the time of day that accessions are responding to WL. Each line is the average response score for each accession in a given module. The number of genes within a module are provided in parentheses. (b) Enriched Gene Ontology (GO) terms for response modules M1, M2, M3, and M8. GO terms are listed on the y‐axis and the negative log of the associated *P*‐value is along the x‐axis. Each bar represents the significance of a given GO term by accession (colors). Colors are grouped by growth responses of the accessions from Figure 1. Terms are grouped by broad descriptor (“parent term”). b.p., biosynthetic process; c.p., catabolic process; m.p., metabolic process.

To more broadly identify the biological processes with time of day‐specific responses across all accessions, we generated a ‘response score’ as the difference between WW and WL expression at each time point for every gene and used these scores to identify global co‐expression response modules. This allowed us to identify 11 time of day specific patterns that now describe the underlying WL responses of all 14 accessions (Figure 4a). For example, M2 contains genes with higher expression under WL in the morning, and lower expression under WL at night. M8 has the opposite pattern, while genes in M3 have higher expression at dawn (ZT1; 1 h after dawn) and at the end of day (ZT13). Based on our analysis of the individual accession phase response groups, we hypothesized that although every accession was found in each module, the underlying function of the genes would be different.

### *B. napus* alters time‐of‐day regulation of carbon fixation and photosynthesis under prolonged WL

We performed GO enrichment on each response score module and accession to determine what biological processes were associated with the time of day‐specific responses. This produced a list of approximately 2700 unique GO terms. We assigned each term into one of 34 ‘broad descriptors’; similar to a parent term (Table S3; ResponseScore_GO_Terms tab). Three out of the 14 accessions were enriched in abiotic stress suggesting that the majority of accessions had adjusted to the prolonged water limitation and were not showing signs of stress, consistent with the phenotyping data. Most accessions (12 out of 14) were enriched in carbohydrate metabolism, carbon fixation, and/or sulfur‐related processes (Figure 4b and Table S3 ‘ResponseScore_GO_Terms’ tab). Carbohydrate‐related terms involved in negative regulation or catabolism of carbohydrates were found in M3 (higher under WL at the beginning and end of day) and were enriched in Al and St (Figure 4b) while Ca was enriched in the same process in M8 (up at night). Genes involved in carbohydrate biosynthesis had more diverse patterns and were found in M1 (up before and after dawn; St), M2 (up during the day; Gr and Se), M3 (up at the beginning and end of day; Mu and St), M7 (down at night/predawn; Ab), M8 (up at night; Gr) and M9 (up at night; Da and Gr). Interestingly, Mu and St were enriched for both negative and positive regulation of carbohydrates in the same module (M3), suggesting that these accessions are turning over carbohydrates early morning and at the end of the day.

From our phenotyping color analysis, we did not see any indications of reduced photosynthesis consistent with plants adjusting their growth to accommodate limited water availability. However, we did find that several accessions (Ab, Br, DH20, Gr, St, Yu) were enriched for changes to photosynthesis (Figure 4b) on the transcriptome level. While some accessions tended to be enriched for terms involved in light sensing (Br‐M5; DH20‐M8; St‐M3 and M8), others were enriched in the light/dark reactions (Ab‐M1), stomatal closure (Ab‐M1 and M8; St‐M1 and M3;Yu‐M9), or had a few terms enriched in chlorophyll metabolism (Gr‐M2; St‐M1, M3 and M8; Yu‐M6). We noticed that although some accessions were enriched in the same terms as another (Ab, Ca, St, Yu), the modules their genes were enriched in differed, implying unique regulation strategies in response to prolonged WL (Figure 4a,b; Table S3). For example, while Ab and Yu were both enriched for stomatal closure (GO: 0090332), Ab's genes were enriched in M8 (higher WL expression in the evening and night) while Yu's were in M9 (little to no difference during the day but higher at night). Similarly, both Ab and St were enriched for the same term involved in light/dark reactions and carbon fixation but had opposing WL responses at the end of day, with Ab (M1) maintaining lower WL expression toward the end of the day and ramping back up at night while St (M3) had higher expression at the end of the day and came back down at night (Figure 4a).

**Figure 5:**
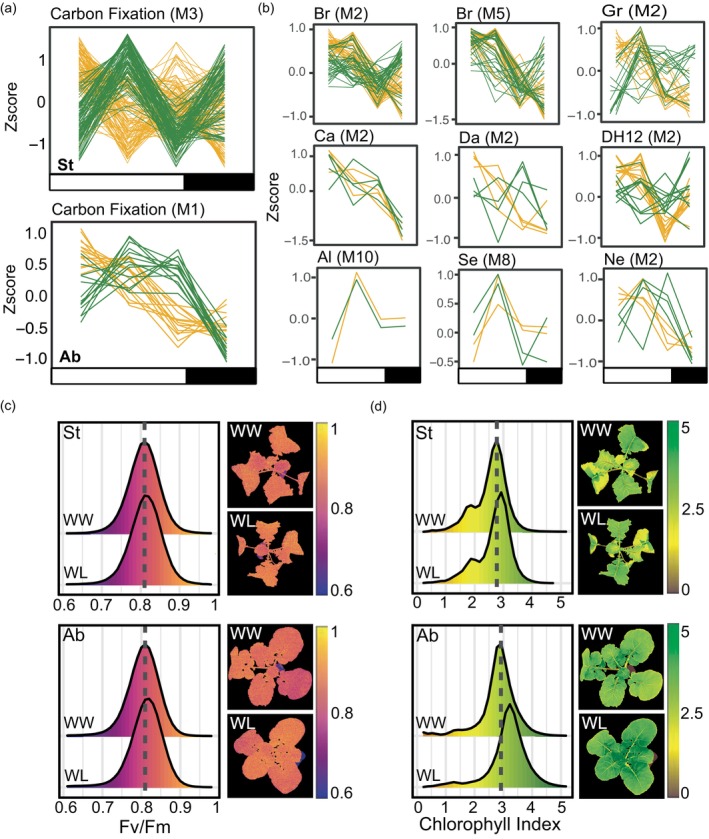
Temporal modifications in transcript levels of enriched photosynthesis and carbohydrate genes and no change in net photosynthetic efficiency under prolonged WL in St and Ab. (a) Expression profiles of WL (yellow) and WW (green) genes enriched in carbon fixation. Bars under the panels indicate lights on (white) and lights off (black). Each line is a gene in a given module which is listed in parentheses. (b) Gene expression of the carbon fixation genes from (a) in accessions that were not enriched in this pathway. Accessions are grouped by growth phenotypes with relatively large (top row), intermediate (middle) and low differences in growth. (c) Efficiency of photosystem II (Fv/Fm) in St (top) and Ab (bottom) under WL or WW for 3 weeks. No significant difference in mean Fv/Fm from WW is seen under WL in either St or Ab (*P* = 0.23 and *P* = 0.19, respectively). (d) Chlorophyll Index (CI) in St (top) and Ab (bottom) under WW or WL for 3 weeks. Significant increase in mean CI is seen under WL in Ab (*P* < 0.0001) but not in St (*P* = 0.05).

As the time of day‐specific WL responses (response scores; Figure 4a) are the difference between WL expression and WW expression at a given time point, there are multiple expression patterns that could lead to a similar response score pattern. To examine the expression patterns themselves we chose two accessions (St; relatively large reduction in growth and Ab; intermediate reduction in growth, Figure 1) that differed in their temporal responses for photosynthesis (Figure 5a). We noticed that, in general, St was dramatically altering expression of carbon fixation‐related genes under WL, where the treatment responses were flipped (antiphase). In contrast, Ab showed a more subtle difference and appears to be maintaining a similar expression pattern overall only slightly up before/after dawn and ramping down expression midday/evening (Figure 5a). We wondered if other accessions had similar WL responses and if the responses differed by growth group (Metadata; GrowthPhenotypeGroups tab). To explore this, we examined the expression of the genes from the carbon fixation enriched process in Ab and St (Figure 5a) that were WL‐responsive in the other accessions (Figure 5b). Similar to St and Ab we see that WL expression was higher early in the morning in the large (Figure 5b; top row) and intermediate (Figure 5b; middle) growth groups and mixed for the low growth group (Figure 5b, bottom), with most of the genes falling into M2 (up in the morning and down at night). Interestingly, we found that the low‐growth group had few WL‐responsive genes while accessions with larger differences in growth had several WL‐responsive genes involved in carbon fixation, despite the lack of significant enrichment.

We were curious if the genes in Ab and St (GO:0015977; carbon fixation) were shared or if there was evidence for homoeolog divergence like we found with our list of sugar‐responsive genes (Figure 3c). While both accessions were enriched in the *PLASTID ISOFORM TRIOSE PHOSPHATE ISOMERASE* ortholog (At2g1170), Ab was enriched for a BnA copy (M8) while St was enriched for a BnC copy (M3). We also found divergence in the time of day that enriched homoeologs altered expression within a single accession (St), where a BnC copy of the ortholog *D‐RIBULOSE‐5‐PHOSPHATE‐3‐EPIMERASE* (At5g61410), was enriched in M3 while the BnA copy was enriched in M1. This was not the case for other overlapping orthologs such as *TRANSKETOLASE1* (At3g60750) that have the same responsive homoeolog. Overall, based on the expression patterns of carbon fixation‐related genes (Figure 5a) and other photosynthesis‐related processes (Figure 4b), we find that Ab and St show unique diel regulation of photosynthesis under WW and WL conditions. This led us to wonder if there was a measurable difference in photosynthesis across the entire day or if the time of day‐specific shifts in expression resulted in the same net photosynthesis between treatments.

### *B. napus* accessions maintain net photosynthesis and chlorophyll content under WL

To capture aspects of photosynthesis outside of transcript‐level responses, we performed a follow‐up phenotyping experiment on a subset of accessions with differential growth responses. We grew these lines under the same 20% WL as done in the original experiment and captured photosynthetic related metrics including dark‐adapted photosystem II efficiency (Fv/Fm), chlorophyll index (CI), total chlorophyll content, and stress‐related indices (NPQ: non‐photochemical quenching; NDVI: Normalized Difference Vegetation Index). Images were taken weekly at ZT8 and were analyzed with PlantCV (https://github.com/danforthcenter/ktgreenham‐brassica‐drought‐paper). Leaf tips were collected at the end of the experiment at ZT6 to quantify chlorophyll levels.

Consistent with the original experiment, plants exhibited few signs of stress and maintained high levels of photosystem II efficiency and CI (Figure 5c; Figure S6) under WL, particularly at day 21 (Figure S6). NPQ and NDVI also remained similar between WW and WL treatments throughout the experiment (Figure S7). Accession Ab (intermediate reduction in growth; Figure 1) had higher average chlorophyll index (CI) under WL (Figure 5c; *P* < 0.0001), but St (relatively large reduction in growth; Figure 1) showed no difference in average CI between WW and WL plants after 3 weeks (Figure 5c, *P* = 0.05). Surprisingly, we found no differences in extracted chlorophyll content in any accession (Figure S6c). This could be due to lower content in the leaf tips compared with the more basal tissue. It could also be due to components other than chlorophyll, such as nitrogen, reflecting in the same CI region (CI_Red‐Edge_; Clevers & Gitelson, 2013). Regulation of nitrogen and sulfur pools is particularly critical for *Brassica* crops due to the production of nitrogen and sulfur‐rich glucosinolates (GSLs). While GSLs are best known for their role in biotic defenses, more recent evidence indicates that they may also be important for abiotic stress responses including drought (Chowdhury, 2022; Hornbacher et al., 2022; Salehin et al., 2019).

### Accessions are altering regulation of sulfur and GSL metabolism in response to prolonged WL

**Figure 6:**
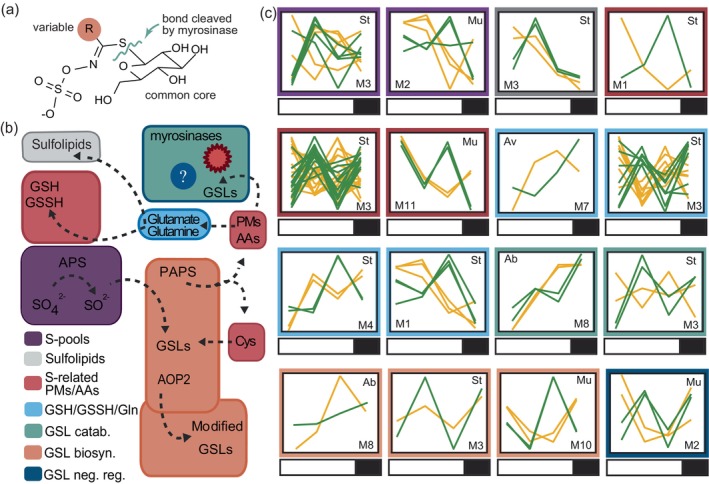
*B. napus* accessions exhibit altered regulation of sulfur metabolism and glucosinolate (GSL) genes at different times of day in response to WL. (a) A model of a generalized GSL which is composed of three parts; the thio‐glucose moiety, a sulfated thio‐hydroximate, and a R (variable) group derived from an amino acid precursor. The conical view of GSL degradation requires GSL specific enzymes known as myrosinases. (b) Simplified schematic of the interaction between sulfur and GSL metabolism. APS (Adenosine 5′‐phosphosulfate) reduces sulfate (${\mathrm{SO}}_{4}^{2-}$) to sulfite (${\mathrm{SO}}_{3}^{2-}$). PAPS (3′‐phosphoadenosine 5′‐phosphosulfate) sulfonates precursor GSLs before they are further modified by 2‐oxoglutarate‐dependent proteins such as AOP2. Sulfur is also allocated toward primary metabolites (PMs) such as glutamate/glutamine that can combine with cysteine to produce glutathione (oxidized: GSSH; reduced: GSH); amino acids (AA) such as Cysteine (Cys), and sulfolipids (ex: sulfoquinovose‐diacylglycerols; SQDGs). (c) Expression patterns of genes enriched in modifying sulfur pools (purple), GSL biosynthesis (peach), and reduction of GSLs (aqua; dark blue), glutamate/glutamine (light blue) and sulfolipids (gray) as indicated by colored boxes. Each line is the z score of a single enriched gene. Bars indicate lights on (white) and lights off (black).

Our transcriptomic analysis revealed four accessions (Ab, Av, Mu, St) that were enriched in sulfur and GSL metabolism (Figure 4) that include fixation of inorganic sulfide (ex: *O‐ACETYLSERINE (THIOL) LYASE (OAS‐TL) ISOFORM A1*), synthesis of sulfur‐related amino acids (ex: *CYSTEINE SYNTHASE D1*) primary metabolites (ex. *GLUTAMINE SYNTHETASE 2*), GSL biosynthesis (SUPERROOT1), and negative regulation (SDI1) or catabolism (*BETA GLUCOSIDASE 26*) of GSLs (Figure 6a). A full list of over‐enriched sulfur‐related genes with their corresponding Arabidopsis ortholog can be found in Table S3 in the “AllSulfurTerms” tab. To explore modifications in diel regulation of WL genes involved in this pathway, we first grouped genes by the parts of sulfur/GSL metabolism they were enriched in (Figure 6b; colored boxes). While most accessions were enriched in one or two steps of the pathway, St was enriched in all aspects that we looked at except for negative regulation of GSLs. In general, under WL Mu upregulated expression of sulfur‐related genes early in the morning (Figure 6c; purple, red, dark blue) while St was more variable. Ab and Av had little enrichment overall but reinforce our previous findings that accessions enriched in similar pathways (or in this case, a specific step of a pathway) tend to have differential time of day expression patterns under WL implying unique regulatory strategies. For example, Ab and Mu are both enriched in genes involved in GSL biosynthesis (peach) but the expression patterns are quite different. While Ab is ramping up throughout the day and peaking just before night in WL (M8), Mu expression is highest at night/dawn (M10) under WL.

We noticed that St had a large proportion of its enriched genes in M3. This module had enrichment in multiple steps of the pathway including aspects of primary sulfur metabolism and GSL catabolism. We were curious if any of these genes were found in multiple copies, and if so, what level of homoeolog expression variation was present. The majority of the sulfur metabolism and GSL catabolism genes were expressed as a single copy, with two exceptions. A TF well known for its' importance in aliphatic GSL biosynthesis (*MYB28*), and an atypical myrosinase (enzymes that catabolize GSLs; *BETA GLUCOSIDASE 33*; *BGLU33*), were expressed in multiple copies but only one homoeolog was WL‐responsive. We looked at all accessions that had at least one responsive copy of *MYB28* and/or *BLGU33* and found variation in which accessions have at least one responsive copy, the number of homoeologs that are responsive, and which homoeolog is responsive among the accessions. This was most apparent in *BGLU33* which was responsive in seven accessions with one (St), two (Br, DH12, Mu, Se), or all three (Gr, Ne) expressed homoeologs called WL. Of those that had two responsive copies, most had one BnA and one BnC copy (Br, DH12, Se) while Mu had two BnA copies. This trend was consistent in the *MYB28* ortholog with accessions having one (Ab, St), two (Br), or four (Mu) of the six expressed homeologs called WL‐responsive. Overall, this transcriptome data suggest that these accessions have unique time of day and subgenome/homoeolog specific sulfur re‐allocation strategies under WL.

### Temporal variation in GSL content in a spring and winter oilseed

We found that St (rapeseed; Canola) had the most enrichment in primary sulfur metabolism and was also enriched in GSL degradation. We hypothesized that St may be allocating sulfur toward primary metabolites and away from GSLs, while other accessions like Mu (a winter variety) had relatively more enrichment in GSL metabolism including biosynthesis. Because GSLs are both nitrogen and sulfur‐rich, and because sulfur is a critical component of chloroplasts (i.e., sulfolipids), alterations to GSL levels may contribute to the ability of these accessions to maintain photosynthetic performance.

To determine whether the GSL metabolites were being altered under WL conditions, we performed a prolonged WL metabolomics experiment with St (relatively large reduction in growth; Figure 1) and Mu (relatively small reduction in growth) under similar conditions to the previous two experiments (Experimental Procedures). We measured photosynthetic rates throughout the experiment to ensure similar responses to the previous two experiments and observed similar photosynthetic rates of WW and WL plants throughout the 28 day experiment (Figure S8). After 4 weeks of WL, we collected tissue punches at the tip of the leaves at ZT4, 12, and 20 for metabolomics and a single time point collection at ZT7 for chlorophyll content. We also collected whole leaves at the end of the experiment to quantify total nitrogen and sulfur content.

**Figure 7:**
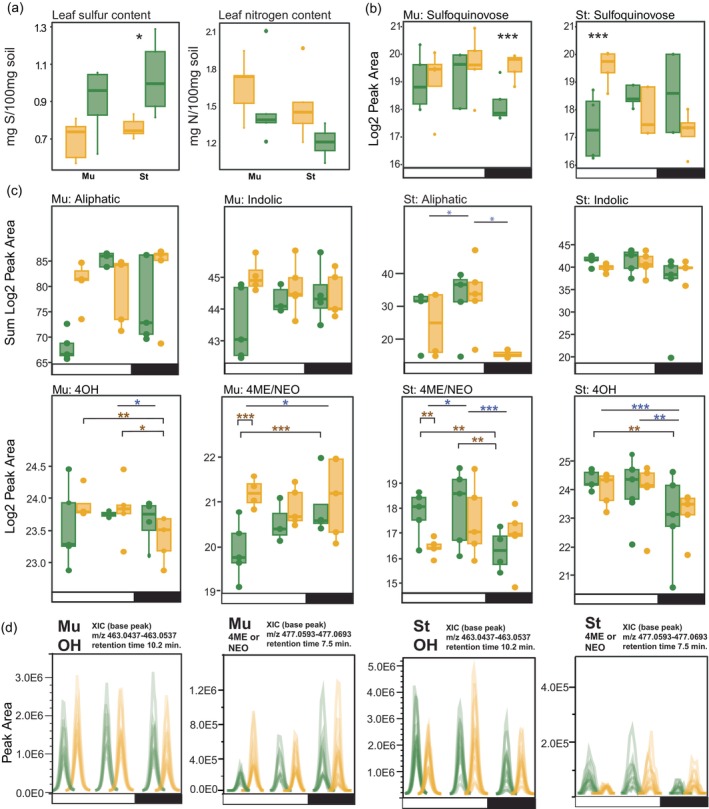
Differential time of day accumulation of glucosinolates (GSLs) in a spring (St) and winter (Mu) oilseed cultivar. (a) Total sulfur and nitrogen content in the sixth leaf of St and Mu collected at ZT7. (b) Sulfoquinovose content (log Peak area) in Mu and St. (c) GSL content (log Peak area) under well‐watered (green) and water‐limited (yellow) conditions at ZT4, 12, and 20 (Zeitgeber; hours after lights on). (d) Traces for Extracted Ion Chromatograms (XICs) for an isomer that is either 4ME or NEO and 4OH. Each line is the unnormalized trace for each LCMS injection for a given feature. Bars indicate lights on (white) or lights off (dark). GSL abbreviations are listed above each panel. 4ME/NEO, 4‐methoxyglucobrassicin/neoglucobrassicin; 4OH, 4‐hydroxyglucobrassicin. Asterisks denote significance (*P ≤ 0.05; **P ≤ 0.01, ***P ≤ 0.001) and are colored by time of day alone differences (blue) or interactive effects between time of day and treatment (brown).

We found that St had significantly less total leaf sulfur content and is trending toward more nitrogen under WL (Figure 7a). This is consistent with what we would expect from this accession as it is a rapeseed cultivar (Scarth et al., 1988) that has been bred for low levels of GSLs (less than 30 μmol g^−1^ dry, defatted meal; Ton et al., 2020). Interestingly, we found significant differences in sulfur‐related compounds between Mu (winter oilseed) and St (spring oilseed) that were time of day‐dependent. Although both accessions had similar levels of sulfur in the leaf in WW (Figure 7a) St had significantly less sulfur in WL and was trending toward higher nitrogen content in WL. Sulfoquinovose (SQ; Figure 7b), a critical component of sulfolipids (e.g., Sulfoquinovosyl diacylglycerols; SQDG's) and part of the thylakoid membrane, increases at night under WL in Mu while St increases in the morning. While both accessions have similar levels of total indolics (Figure 7c), Mu has approximately twice the amount of aliphatics. This is especially apparent at night under WL conditions where St has very low levels of aliphatics (Figure 7c; Figure S9a).

We then looked at individual GSLs and found that the largest differences were in the two indolics we detected (4OH and an isomer that is either 4ME or NEO; Figure 7c). Again, we see unique patterns among the accessions where Mu is increasing 4ME/NEO content in the morning under WL (*P* < 0.000) while St is decreasing content at this time (*P* = 0.008). Under WW, Mu appears to be slowly ramping up content throughout the day (4ME/NEO) and into the night while St has the opposite response. 4OH is somewhat more consistent for both accessions with a minor reduction under WL at night in Mu (*P* = 0.050) while St shows no treatment effects for 4OH. We also inspected variation across the LC–MS run itself to account for any technical variation or matrix effects that might contribute to these differences. While there is some variation between injections within a given treatment/ZT (Figure 7d), peak areas are largely similar as seen in the overlap of peaks between biological/technical replicates.

Methionine‐derived aliphatics (NAP, PRO, SIN, GJV) were surprisingly inconsistent for St, with two of these (SIN; GJV) being entirely below our limit of detection (LOD; 1.0E4 Peak height or 13.3 Log Area; Figure S9a) while the other two (NAP; PRO) were rarely above our limit of quantification (LOQ; 5.0E4 Peak height or 15.6 Log Area; see Experimental Procedures). Although Mu does not appear to have treatment effects for any of the aliphatics, we did see that similar to 4ME/NEO, the WW samples increase significantly at the end of the day (NAP: ZT4–ZT12, *P* = 0.008; ZT4–ZT20, *P* = 0.003; PRO: ZT4–ZT12, *P* = 0.002). This trend appears similar for SIN but we were unable to capture significant differences. A single aromatic GSL (NAS), derived from phenylalanine, has significantly higher levels of ZT4 but only in Mu, although both accessions exhibited a treatment effect that was not specific to a particular ZT (Figure S9b).

## DISCUSSION

To assess how diverse crop types of *B. napus* alter transcriptome regulation of key biological processes after a prolonged period of water limitation, we imposed an 80% reduction in available water on 16 accessions over a 4‐week period and captured phenotypic responses throughout the experiment using a high‐throughput phenotyping platform (Bellwether Phenotyping Facility, Saint Louis, MO). Our growth curve models showed that all accessions exhibited a reduction of above‐ground growth with differences in the magnitude of change between well‐watered (WW) and water‐limited (WL) conditions. We found that all accessions appeared photosynthetically healthy and were not exhibiting signs of stress, indicating that accessions were adjusting to the lack of water early on. In Arabidopsis, water limitation has been shown to reduce leaf emergence, leaf number, and biomass (Dudley, 1996) with no significant effect on net photosynthesis or dark respiration (Hummel et al., 2010). Despite the lack of visible signs of stress, we found quite dynamic changes in the transcriptome.

### Differential contribution of subgenomes to expression dynamics in response to WL

The 24‐h time course RNA sequencing experiment on all accessions provided a unique perspective on the diel regulation of the transcriptome under WW and WL conditions. We found that the majority of WL‐responsive genes were unique to few accessions that were not due to crop type. However, there was a much higher proportion of shared genes when converting the *B. napus* genes to their Arabidopsis ortholog suggesting an overlap in functional responses. What was especially striking was the extensive variation in which homoeolog(s) among the two parental subgenomes were responding under prolonged water limitation. This was evident within and between subgenomes, with some accessions having a single significantly differentially expressed homoeolog from one subgenome, while both homoeologs were significant in other accessions. While the BnC subgenome (*B. oleracea*) had higher expression overall, our phase analysis showed that the BnA contribution (*B. rapa*) differed by time of day (phase) and/or by accession. The contribution of each subgenome to the phase groups varied across accessions. Some accessions, such as Ca, Av, and Br, showed a stronger bias toward representation from BnC, while others, including Ab, Gr, and Yu, exhibited a more balanced contribution. Notably, there were phase group‐dependent differences in subgenome contributions within individual accessions. For example, in Mu, the +6 h phase group displayed a greater contribution from BnC compared with BnA, unlike the other groups. Again, we did not identify any clear patterns correlating with crop type. However, a more time‐resolved dataset may be necessary to obtain more accurate phase values and thoroughly assess these relationships. In general, these results indicate that both subgenomes are contributing to the overall response, but the magnitude of this contribution depends on the time of day and the accession.

Variation in the responsive subgenome and homoeolog copy was consistent across multiple pathways including sugar responsiveness, carbon fixation, sulfur allocation, and GSL catabolism that likely resulted in differences in GSL content. These findings are consistent with previous quantitative trait loci (QTL) and GWAS studies examining variation in GSL content. QTL and GWAS analysis combined with transcriptomics have associated variation in homoeolog expression of the Arabidopsis orthologs of *MYB28*, *MYB29*, and *GLUCOSINOLATE TRANSPORTER‐2 (GTR2)* with altered levels of GSL content in *B. napus* seeds (Wei et al., 2019). Genomic deletions of *MYB28* homoeologs are found in *B. napus* lines with low GSL levels (Harper et al., 2012) consistent with the role of this R2R3‐MYB TF in regulating aliphatic GSLs (Gigolashvili et al., 2007; Sønderby et al., 2007). Multiple orthologs of the bidirectional GSL transporter GTR2 have been identified in *B. napus* that contribute differentially to GSL seed content. In addition to variation in protein sequence, altered tissue‐specific expression exists among the homoeologs (Tan et al., 2022). These data highlight the inherent complexity of polyploid crops where multiple homoeologs contribute to a trait with differential regulation at the tissue level and following abiotic stress (Calderwood et al., 2021).

The widespread variation in homoeolog responsiveness across *B. napus* accessions is surprising for a relatively young crop (approximately 7800–12 000 years old; Chalhoub et al., 2014). This variation might be partly explained by the ease with which its parental species, *B. rapa* and *B. oleracea*, hybridize and form new polyploids through spontaneous chromosome doubling (Song et al., 1995). Recent studies have revealed significant gene content variation among different morphotypes of these species (MacNish et al., 2024), with some research indicating that nearly 20% of genes in *B. oleracea* are affected by presence/absence variation (Golicz et al., 2016). Given this inherent variability and the potential to form new polyploids from different morphotype combinations, it is plausible that *B. napus* has multiple origins. While one study supports this hypothesis (Allender & King, 2010), more recent research has yielded conflicting results (Allender & King, 2010; An et al., 2019). Furthermore, introgression from diploid species into *B. napus*, which can yield meiotically stable tetraploid lines despite initially forming sterile triploids, may be another source of variation among cultivars (Cao et al., 2023). Additional variation may arise from post‐polyploidization processes, such as fractionation (loss) and homoeologous exchanges, which can shuffle and modify regulatory regions (Wendel et al., 2018). Rearrangements in the regulatory architecture, including promoter and enhancer sequences, could alter TF binding sites and contribute to the divergence in homoeolog expression patterns and stress responsiveness. In *B. rapa*, divergence in circadian regulation of paralog expression has resulted in instances where one copy retains a more ‘Arabidopsis‐like’ expression pattern that could be predicted based on the shared presence of conserved non‐coding sequences (Greenham et al., 2020). We would expect a similar level of regulatory variation in *B. oleracea* that would contribute to additional variation in *B. napus*. The lack of information on the specific progenitors that gave rise to the accessions in this study makes it challenging to pinpoint the sources of variation.

### Accession‐specific phase changes in photosynthesis‐related genes under WL

To compare the transcriptomic responses across accessions we generated a response score for every gene and clustered them based on their response patterns. This resulted in 11 distinct time of day‐dependent WL response patterns among the accessions. We found all accessions represented in each cluster but the biological processes were quite distinct. Even among accessions with similar growth responses to the WL condition (St and Av), we found very little overlap in the diel responses for similar pathways. We examined genes involved in photosynthesis and found dramatic phase changes across several accessions. Two accessions, St and Ab, with large growth differences, tended to have high WL expression of genes involved in carbon fixation in the morning with decreasing expression throughout the day. This is in contrast to WW conditions where peak expression occurred from ZT7 to ZT13 in Ab and at ZT7 and again at ZT19 in St. Across accessions the expression patterns for genes involved in carbon fixation varied dramatically with some showing minimal phase changes and others exhibiting an earlier phase of several hours. Although we observed these dynamic temporal changes in genes involved in photosynthesis, our CropReporter follow‐up experiment confirmed that these accessions were photosynthetically healthy under WL conditions. The variation in diel regulation of photosynthesis‐related genes in similarly responding genotypes suggests that these accessions adjusted to limited water using alternate regulatory strategies. Time of day‐dependent transcriptome responses to abiotic stress have been shown in other plant species (Bonnot et al., 2023; Greenham et al., 2017; Robertson et al., 2022; Wilkins et al., 2010), confirming the complexity of the regulatory network. In this study, we demonstrate additional complexity across genotypes that exhibit distinct expression pattern responses for photosynthetically healthy plants. This raises several questions about the timing of these shifts in expression during water limitation and whether they contribute to adaptation to the new conditions.

### WL alters diel regulation of sulfur and glucosinolate metabolism

*B. napus* allocates sulfur and nitrogen supplies to glucosinolates (GSLs), specialized metabolites that are important for biotic (Barco & Clay, 2019) and abiotic (Salehin et al., 2019; Zhu & Assmann, 2017) defense. The balance between sulfur and nitrogen is critical for optimizing yield (Jamal et al., 2010; McGrath & Zhao, 1996). The enrichment of GSL‐related terms for several accessions in our WL conditions led us to examine whether the metabolites were affected. We found evidence for unique diel regulation among the two accessions in both sulfoquinovose (SQ) and GSLs. Although both accessions had similar amounts of sulfur content in the leaves, Mu (low reduction in growth under WL) had significantly higher SQ under WL at night while St (high reduction in growth under WL) was significantly higher in the morning. We hypothesized that these temporal differences in sulfur pools would lead to time of day‐specific patterns of other sulfur‐intensive metabolites such as GSLs. We found opposing patterns of an indolic GSL between the accessions and as compared with SQ in WL conditions. While 4ME/NEO abundance increased significantly in Mu in the morning, in St 4ME/NEO abundance decreased in the morning suggesting distinct differences in sulfur allocation strategies under WL. In WW conditions Mu displayed an increase in GSL content (NAP, PRO, SIN, 4ME/NEO) throughout the day, while St showed little treatment response or decreased content (4ME/NEO), again highlighting alternate regulatory strategies among the accessions. Variation in GSLs is likely to be affecting available sulfur pools, thereby affecting primary metabolite pools (Cystine; Glutathione) and nitrogen use efficiency (Jamal et al., 2010). Further, as auxin and indolic GSLs such as 4ME/NEO are both derived from tryptophan, there is potential competition between these two pathways for sulfur reserves. Alternatively, there may be cross‐talk between these pathways where one pathway acts to mediate the other (Salehin et al., 2019; Zhu & Assmann, 2017) to maintain plant growth under WL (Ludwig‐Müller et al., 2000). Further study into the relationship between diel regulation of sulfur allocation and abiotic stress response is needed to understand the tradeoffs in time of day‐specific changes to sulfur pools.

In the upcoming years, global crops will be subjected to more extreme temperatures including variation in water availability. To avoid reductions in crop yields, we must develop varieties more tolerant to these challenging environments. However, we found substantial variation in which homoeolog copy between parental subgenomes was most responsive and in the time of day they were altering expression under WL, further challenging our ability to identify good breeding targets. Regulatory regions may be more effective and conserved targets for improving crop tolerance under the effects of climate change.

## EXPERIMENTAL PROCEDURES

### Plant materials and growth

Seeds from *B. napus* accessions were germinated in soil in a growth chamber for 5 days before transplanting 16 seedlings per accession per treatment into 4 inch wide, 0.47 L pots (Pöppelman TEKU, Lohne, Germany). Plants were germinated and grown in MetroMix360 soil mixed with Osmocote Classic 14‐14‐14 fertilizer (Everris NA Inc., Dublin, Ohio, USA). Transplanted seedlings were randomly placed in the Conviron (Controlled Environments Limited, Winnipeg, Canada) growth house of the Bellwether Phenotyping Facility (Donald Danforth Plant Science Center, St. Louis, MO). Growth house conditions were set to 22°C/18°C day/night temperatures, 14 h day lengths, 40% relative humidity, and 400 μmol m^−2^ sec^−1^ light intensity. Plants were equally separated into two treatments for the duration of the experiment: 100% watering (Well‐Watered, WW) and 20% watering (Water Limited, WL). Target watering weights were determined as described in Fahlgren et al. (2015). All pots were watered once to 100% capacity on the first day of the experiment and then weighed twice per day to maintain a target water weight through day seven on the Bellwether Phenotyping Facility. Starting on day eight, WL plants were allowed to dry down to 20% capacity and maintained at 20% capacity until day 14 (7 days after water limitation treatment began). At this point, all watering amounts were increased by 10% for an additional 14 days to compensate for plant biomass. Plants were automatically weighed and watered twice daily to maintain treatment water levels.

### Image capture

Detailed descriptions of the Bellwether Phenotyping Facility and associated LemnaTec imaging systems (LemnaTec Scanalyzer 3D) are available in Fahlgren et al. (2015). On each day of the experiment, all plants moved from the controlled environment growth house to the imaging loop via a conveyor belt. The imaging loop contains two imaging chambers with visible light (Red Green Blue, RGB) and near‐infrared (NIR) cameras (Basler AG, Ahrensburg, Schleswig‐Holstein, Germany). Two side view images from 0° and 90° angles were captured each day by the RGB and NIR cameras. Zoom levels were adjusted two times during the experiment as the plants grew. A total of 59 850 RGB and 59 848 NIR images were captured during the experiment. Images are available here: https://datacommons.cyverse.org/browse/iplant/home/shared/danforth_center/Ricono_et_al_Brassica.

### Image analysis

Images captured by the Bellwether Phenotyping Facility were analyzed with PlantCV v3.14. Typical PlantCV image analysis workflows involve segmenting the plant from the image background by creating a mask and then using PlantCV analysis functions to extract data from the plant image. All PlantCV scripts used for image analysis are available in the public GitHub repository: https://github.com/danforthcenter/ktgreenham‐brassica‐drought‐paper. Because some *B. napus* leaves can have a purple hue or become yellow as they age, simple segmentation methods on RGB images often fail to capture the whole plant. To segment plants in the RBG images, the PlantCV naïve bayes multiclass training module was used to classify pixels as “healthy,” “senesced,” or “background.” To train the naive Bayes classifier, the RGB values of pixels were sampled across multiple images to create a training dataset of 984 pixels belonging to each category. Pixels were chosen from green and purple leaves for the “healthy” category, from yellowing leaves for the “senesced” category, and from all non‐plant aspects of the image for “background.” The trained classifier was used to segment plants from all images and calculate the percentage of pixels in each plant that were classified as “senesced.” After segmentation, a rectangular region of interest was positioned around the plant to further eliminate noise. The PlantCV “analyze_object” and “analyze_color” functions were used to measure the size of the whole plant and the color characteristics of the healthy and senesced leaves in each plant image. For all RGB images, a separate PlantCV image analysis workflow was created for each zoom level. Resulting pixel values were converted to physical measurements (i.e., cm and cm^2^) using a custom R script to combine the standardized data (Fahlgren et al., 2015). Plant area values from 0° and 90° angle images of each plant were summed.

For NIR images, only images from day 14–day 26 were used so that the NIR signal data were measured from images at the same zoom level since the zoom configuration impacts NIR values. Masks generated in the RGB workflow were resized and repositioned to segment the plant in the NIR image. NIR pixel intensities were extracted and the mean NIR signal was calculated using the “analyze_nir_intensity” function in PlantCV.

### Statistical analyses

Data curation and statistical analyses were done in R (R Core Team, 2020). All R scripts related to the phenotyping analysis are available at the public GitHub repository: https://github.com/danforthcenter/ktgreenham‐brassica‐drought‐paper. Plant area was modeled per each accession and treatment group using a Bayesian Hierarchical growth model. We used a Bayesian model for the flexibility in accounting for heteroskedasticity and hypothesis testing. Our data variables were plant area (cm^2^) and time which was expressed as days after start (days). We used a Gompertz growth model ($y=A^{-B^{-\mathrm{Ct}}}$) to estimate the model parameters where *A* is the asymptote, *B* is the inflection point, *C* is the growth rate of the growth curve, and *t* is time; and we used a logistic model to model the variance over time using the parameters *subA* (the asymptote on variance per treatment group), *subB* (the inflection point for the increase in variance), and *subC* (the rate at which variance increases). This was a distributional model using the Student's T distribution and thus includes priors on the growth model parameters, the variance model parameters, and the degrees of freedom. We used lognormal priors for each estimated parameter in our growth model (*A*, *B*, *C*) and in the sub‐model for variance (*subA*, *subB*, *subC*). For the asymptote parameter, we used a lognormal distribution with mu = 130 and sigma = 0.25. For the inflection point parameter, we used a lognormal distribution with mu = 15 and sigma = 0.25. For the growth rate parameter, we used a lognormal distribution with mu = 0.25 and sigma = 0.25. In the variance sub‐model, we used lognormal priors with a sigma of 0.25 centered at 20, 10, and 3 for *subA*, *subB*, and *subC* respectively. These thick‐tailed lognormal distributions were chosen to make sure the data could pull away from the prior if necessary and because plant area must be positive. We used Student's T prior distributions for smooth terms of variance with a mean at 0, standard deviation of 5, and 3 degrees of freedom and a gamma (2, 0.1) prior to the degrees of freedom. All priors and further details and the code to implement our models are on Github: https://github.com/danforthcenter/ktgreenham‐brassica‐drought‐paper. Prior predictive checks were conducted using the plotPrior function from the pcvr R package (Sumner et al., 2023) (Figures S10 and S11). These models were fit in R using the brms (brms version 2.20.4, cmdstanr version 2.34.0) and pcvr packages (version 0.1.0). We used two chains, each with 1000 post‐warmup draws. Our bulk and tail effective sample sizes based on MCMC range from 700 to 2200 with an average neff ratio of 0.57 and no neff ratios below 0.29. Finally, our Rhat values show good convergence with no Rhat values above 1.0106 for model parameters (Table S1). For details on our hypothesis testing see the Results and Discussion sections and Table S1.

### Tissue collection and RNA sequencing

After 2 days (21 treatment days) on the Bellwether Phenotyping Facility, leaf tissue was collected from the apex of the youngest leaf on each plant. Tissue was collected at ZT (Zeitgeber; hours after dawn) 1, 7, 13, and 19. For each accession, tissue was collected from three replicates of each treatment at each time point. RNA was isolated using the MagMAX Plant RNA Isolation Kit (Thermo Fisher Scientific, Waltham MA). RNA was submitted to the DOE Joint Genome Institute for library preparation and sequencing as part of a Community Science Program grant CSP504418. RNA sequencing was performed on a Novaseq S4 with PE150. Data are available through the NCBI GEO database (GSE261928).

### RNA sequencing processing

Raw reads were mapped to a concatenated reference consisting of *B. rapa* R500 v1.2 (Lou et al., 2020) and *B. oleracea* TO1000 (Parkin et al., 2014) genomes. This was accomplished by simply concatenating the source reference FASTA files together. The reads were trimmed and filtered by quality using Trimmomatic v0.33 (ILLUMINACLIP:TruSeq3‐PE‐2.fa:2:30:10 LEADING:3 TRAILING:3 SLIDINGWINDOW:4:25 HEADCROP:14 MINLEN:50), and alignment was performed using HISAT2 2.1.0 (max_intron = 12 000, strandness = RF). Feature counts were created from bam files using the ‘featureCounts’ function from Rsubread R package (‐F GTF ‐M ‐‐fraction ‐s 2 ‐p ‐B ‐C) and normalized using edgeR (R Core Team, 2020). All counts from each sample are compiled into accession‐specific tables and provided on the NCBI GEO database (GSE261928). Genes with no variance in expression across the time series were removed; the remaining genes are referred to as “processed genes” and are also available through the NCBI GEO database (GSE261928). We further filtered out lowly expressed genes by retaining those with a mean log2 FPKM greater than zero at at least one time point. For the pattern detection in DiPALM, gene expression for any missing replicate (Table S3; WLgenes tab) was imputed as the median of the two remaining replicates for that time point. All code for the RNA‐seq analysis can be found in the associated R Markdown file on github https://github.com/GreenhamLab/Bnapus_WL_TranscriptomeTC.

### Expression principal component analysis

Raw expression counts from FeatureCounts of all individual samples was merged with the script ‘combining_featCount_tables.py’ from the Bioinformatic Notebook (Harrington, 2020), the combined counts table was loaded into DESeq2 v.1.26 (Love et al., 2014) under R v3.6.3 (R Core Team, 2020). Read counts were normalized with the variant stabilizing transformation and principal components were calculated with the plotPCA function and visualized with ggplot2 (Villanueva & Chen, 2019).

### SNP calling and principal component analysis

SNPs were called using GATK v4.1.4.1 (Poplin et al., 2018; van der Auwera & O'Connor, 2020) on single replicates of RNA‐seq data for each accession, taken from control treatment plants at ZT1. Sequencing reads were first trimmed using Trimmomatic v.0.33 (Bolger et al., 2014) to remove Illumina TruSeq3 adapters. Trimmed reads were mapped to a concatenated *B. oleracea* acc.TO1000 (Parkin et al., 2014) and *B. rapa* acc. R500 (Lou et al., 2020) reference genome using STAR v2.6.0 (Dobin et al., 2013) with settings “‐‐outFilterMultimapNmax 1” and “‐‐outFilterScoreMin 10.” We used a two‐pass mode to improve mapping performance around splice junctions. Duplicates were marked and read groups were added with Picard v2.25.0 (Broad Institute, 2019). Reads with Ns in the Cigar string, representing reads that span splice events, were split using the GATK v4.1.4.1 SplitNCigar tool. Each accession was processed with the pipeline independently, and genotyped independently with GATK HaplotypeCaller, using diploid settings. Individual gVCF files were combined with CombineGVCFs, and then jointly genotyped with GenotypeGVCFs, resulting in a single VCF file for the 16 samples containing 4 599 922 raw variants. After removing Indels with GATK SelectVariants, 3 439 690 SNPs remained.

A series of variant filters were used on this combined vcf to address possible technical issues related to the sequencing and alignment steps and produce a high‐confidence set of SNPs. These included filters for strand biases and mapping quality (e.g., removing sites with SOR ≥4; FS >60, ReadPosRankSum >10 and <−10, and MQ <30), genotyping quality metrics (removing variants with QUAL <0 and QD <20) and site depth (removing variants with DP <15). The distribution of values for all the filters used on the dataset was used to select thresholds. After filtering, 620 331 SNPs were retained and used for principal component analyses, performed with Plink v1.90b6.16 (Purcell et al., 2007) and visualized in R v 3.6.3 (Team, 2023) with ggplot2 (Villanueva & Chen, 2019).

### DiPALM analysis

DiPALM (Greenham et al., 2020) was used to identify differentially patterned genes for each accession. This pipeline identified sets of genes with either a change of phase (kME), or change in median expression level (Med), across the time course. Two accessions (Qu and Ze) were dropped from downstream analyses as they had strikingly low numbers of kME genes (17 and 16; respectively) and were unlikely to be informative. Due to the missing replicates and the need to impute for the DiPALM analysis, DiPALM calls for the remaining 14 accessions were validated by comparing the number of differentially patterned genes to the number of genes called by single time point analyses for the time points with three replicates. Linear models were run at each time point and accession and the final numbers were compared with the DiPALM calls. Results were largely comparable; for full details see the RMarkdown available at https://github.com/GreenhamLab/Bnapus_WL_TranscriptomeTC. For accessions with missing replicates, we selected the DiPALM calls that overlapped with the single time point analyses.

### Subgenome comparison

To quantify the relative diel contribution of each subgenome (BnA, BnC) to the number of WL genes for each accession, we identified the average phase (peak timing of expression) of each gene and then calculated the phase difference between WW and WL. This resulted in five phase change groups (0 = no change; 6 = 6 h earlier; 12 = 12 h earlier; −6 = 6 h delayed; −12 = 12 h delayed). Within each phase change group, we calculated the proportion of WL genes relative to the total number of WL genes for each accession. We then identified similar phase changes under WL in our DiPALM clusters and ran Gene Ontology (GO) analyses to identify differences in the regulation of enriched biological processes for these clusters. Differentially patterned *B. napus* subgenome homoeologs (kME; Med) were compared to identify the proportion of shared genes that respond to WL among the accessions. For each WL gene, the number of accessions was summed and the proportion was calculated relative to the total number of shared genes from all accessions. Syntenic orthologs in Arabidopsis for each WL gene were also analyzed and the relative proportion calculated as described for subgenome‐specific homoeologs.

### Response score module assignment

To compare WL transcriptomic responses across accessions, we generated response scores by taking the difference between WL and WW expression for every gene at each time point for all accessions. The response scores for all accessions were used in a co‐expression network analysis (Langfelder & Horvath, 2008) to identify global response score modules across accessions. A Gene Ontology (GO) analysis was done for each accession per response module using previously compiled annotations (Greenham et al., 2020) and their corresponding biological process. As the concatenated *B. napus* reference inherits gene annotations from the source R500 and TO1000 references, GO term mappings were generated by associating accessions from the source references to their orthologs in the TAIR10 reference (Arabidopsis) which has functional annotations. In two cases (M5; M9), some accessions had opposing responses within a given module/time point (i.e., some were up in WL and some down at a given ZT). In these cases, the module was further split and GO was run separately. Results of each analysis were compiled and GO terms were grouped and assigned to a “broad descriptor,” analogous to a parent term, to reduce complexity and to aid in interpretation (Table S3; ResponseScore_GO_Terms tabs).

### Plant materials and growth for CropReporter measurements

Six *B. napus* accessions (Ab, Ne, Mu, Av, St, and DH12) were grown in conditions mimicking the original experiment done on the Bellwether Phenotyping Facility. Seeds were germinated on wet Whatman paper to improve germination rates. All but St were allowed to germinate for 3 days; St was given 5 days. Five replicates per treatment and accession were sown into 4 inch diameter, 0.47 L pots (Pöppelman TEKU, Lohne, Germany), filled with equal amounts of a custom Jolly Gardener soil mixture (made as closely as possible to MetroMix360, which was used in the previous phenotyping experiment but was no longer available at the time of this experiment). The accessions were split into three groups of two accessions each, and germination and planting were staggered to allow subsequent imaging during the experiment to occur within a 2 h window at the same time of day for all accessions. Plants were manually watered daily to a specific target weight as described above. Plants were watered to control levels with fertilized water for 6 days after planting before the 20% water‐limitation treatment began at 7 days after planting. After this point, plants were only watered with reverse osmosis water. At 14 days after planting, 7 days after the treatment began, watering amounts were increased by 10% to account for the growing plants, as was done in the previous phenotyping experiment.

### CropReporter imaging

Whole plant chlorophyll fluorescence and multispectral images were captured using the PhenoVation CropReporter (PhenoVation Life Sciences, Wageningen, The Netherlands). Images were captured from the top down, and the distance from the camera to the top of the plants was kept consistent at ~105 cm throughout the experiment. Plants were dark‐adapted for 30 min prior to imaging. We first captured the induction curve of the dark‐adapted plants followed by a 6 min light‐adaptation with actinic light at a light intensity of 400 μmol m^−2^ sec^−1^. The induction curve of the light‐adapted plants was captured as well. More detail is available in the methods described in Lazarević et al. (2021, 2022). Plants were imaged at ZT8 on day 0 (pre‐treatment), and 7, 10, 14, and 21 days after the water limitation treatment began. Images are available on CyVerse: https://datacommons.cyverse.org/browse/iplant/home/shared/danforth_center/Ricono_et_al_Brassica.

### CropReporter image and statistical analyses

Image analysis was done with the open‐source software PlantCV (Fahlgren et al., 2015; Gehan et al., 2017) v3.14.1 (Casto et al., 2021). Data visualization and statistical analyses were conducted in R v4.3.1 (R Core Team, 2020), largely using the pcvr package (Sumner et al., 2023). Values were normalized for plant size. Scripts are available in the public GitHub repository https://github.com/danforthcenter/ktgreenham‐brassica‐drought‐paper.

### Plant materials and growth for physiology and metabolomics

Seeds for *B. napus* accessions Mu and St were imbibed on wet Whatman paper and sown in a 50/50 mixture of Sungro Professional Growing Mix and ProMix BRK in 4.5 × 4 inch circular pots. Soil was dried down at 65°C for 4 days and hand‐mixed intermittently. About 15 plants per treatment were grown for St and Mu. All plants were grown in a Conviron chamber as described above, with slightly higher (~60–80%) relative humidity. Target weights were re‐calculated to account for differences in soil types. All plants were fertilized with 100% field capacity liquid Hoaglands fertilizer upon sowing. All pots were maintained at 80% or 20% field capacity the first week of growth and target weights were increased by 10% thereafter. Water limitation was initiated after 1 week of 100% field capacity watering. Pots were manually weighed daily throughout the experiment to maintain water levels.

### Photosynthetic rate

Photosynthetic rate (*A*) was measured using a Li‐COR LI‐6800F Portable Photosynthesis System (LI‐COR, Lincoln NE, USA). Physiological responses were recorded at ZT4 and ZT12 at least once a week on five replicates throughout the experiment starting from 1 week after WL. Measurements were taken on the first fully unfurled true leaf for the first 3 weeks followed by leaf five (22–27 days after WL) and seven (28+ days after WL) to account for maturation and senescence. Chamber settings matched the environmental conditions of the growth chamber. Li‐COR measurements were as follows: flow rate, 500 mmol sec^−1^; CO_2_ concentration, 400 mmol mol^−1^; VPD, 0.8–1.1 kPa; and leaf temperature, 22°C.

### Chlorophyll content

Chlorophyll content (a; b) was measured for each Li‐COR replicate (*n* = 5 per accession; *N* = 30) from the seventh leaf using a 9 mm (~12–18 mg) leaf punch taken at the top and left of the midvein at ZT7. Each punch was immediately submerged in glass vials containing 2 mL of 80% acetone and shaken overnight in the dark. The following morning, extracts were quantified on a spectrometer (Molecular Devices SpectraMax i3X) in a 2:1 ratio of extract: 80% acetone. Samples were run in triplicate.

### Metabolomics

Metabolomics samples were collected at ZT4, ZT12 and ZT20 from the seventh leaf that was paired with chlorophyll leaves using the same 9 mm core borer as described for chlorophyll content. To avoid rapid metabolite degradation associated with a wounding response, 50 mL Falcon tubes were placed on dry ice and partially filled with liquid nitrogen to create ultra cold reservoirs. Leaf disks were immediately dropped into reservoirs and stored in liquid nitrogen until they could be transferred to individual screwtop tubes with a single 2 mm tungsten carbide bead and stored at −80°C until pickling. “Pickling” involves fully submerging tissue in 40% w/v 100% HPLC grade methanol on dry ice prior to storage for at least 2 weeks at −80°C to slowly denature myrosinases and other enzymes. Tissue was then homogenized using frozen adapters for a Qiagen TissueLyser II and homogenized at a rate of 30 Hz for 45 sec for one to two rounds, or until the tissue was fully homogenized. Samples were subjected to centrifugation at 15 000 rpm and 4°C for 15 min to separate the supernatant from cellular debris. Supernatant was collected and spun once more at 15 000 rpm in 4°C for 5 min before storing at −80°C.

Samples were diluted (1:4) before injection onto a UHPLC (Ultimate 3000, Dionex) ZIC‐cHILIC column (100 mm × 2.1 mm, 3 μm particle size, EMD Millipore Corporation, Billerica, MA) at a flow rate of 1 μL min^−1^. Solvents A (0.1% v/v formic acid in water) and B (0.1% v/v formic acid in acetonitrile) were used as mobile phases for gradient separation. Gradient separation was as follows: 2 min from 98% B, 0.5 min to 88% B, 1 min to 85% B, 2.75 min to 72% B, 0.75 min to 70% B, 2 min to 65% B, 1 min to 55% B, 1 min to 45% B and 98% B maintained for 2 min. The column was equilibrated for 2 min with 98% B prior to the next injection. Samples were detected using a hybrid quadrupole Orbitrap mass spectrometer (Q Exactive, Thermo Fisher Scientific, San Jose CA) with an autosampler and sample vial block maintained at 4°C. Full scan MS (99–1500 *m/z*) were acquired with positive and negative mode using polarity switching. The sheath gas, aux gas, and sweep gas were set to 50, 20, and 1 psi respectively. The capillary temperature was 350°C. The S‐lens RF value was set to 55 (arbitrary units). The Aux gas heater temperature was 300°C. Samples were processed in two batches, one for each accession. Biological samples were run in triplicate to account for technical variation between runs. Data were acquired using Xcalibur™ software version 2.1 (Thermo Scientific). Raw data were converted to .mzML files using MSConvert and targeted peak detection was performed in mzMine 2.53 (Chambers et al., 2012). Visualization of chromatograms was performed using mzMine to assess potential drift in retention times (RTs) and to assign limits of detection (LODs, 2x background) and limits of quantification (LOQs, 5x background) for each GSL. For full details including mass ranges used for extracting features see Table S4. We normalized across technical replicates by summing the relative abundance over a representative negative mode region of each run (Mu: RT 3.20–6.90 and *m/z* 600–1200; St: RT 5.2 to 6.8 and *m/z* 640–1220) and then divided each relative abundance value by the sum of this region for that sample. This value was then multiplied by the average of the representative region across all samples by accession to account for variation across runs. Raw data are available on the Data Repository for U of M (DRUM; https://conservancy.umn.edu/collections/6c548d8b‐0f3a‐4f6b‐b16e‐4154c88136c0).

### Leaf nitrogen content

The entirety of the seventh leaf was collected, weighed, and fully dried for sulfur and nitrogen content. Approximately, 1 g of dry tissue was sent to the Analytical Laboratory at the University of Minnesota's St. Paul campus (https://ral.cfans.umn.edu/).

## CONFLICT OF INTEREST STATEMENT

The authors declare that they have no conflicts of interest.

## Supporting information


**Figure S1.** Percent of plant pixels classified as “yellow” by a Naive Bayes classifier. *Brassica napus* accessions exhibit reduced growth with water limitation (Figure 1). Several accessions on average have a lower percentage of plant pixels classified as ‘yellow’ at the end of the water limitation treatment compared with the well‐watered treatment.
**Figure S2.** NIR signal is higher in most plants in water‐limited treatment, suggesting lower water content than in well‐watered plants. Only NIR reflectance from day 14 to day 26 are shown, when images were taken at a consistent zoom level. Values were averaged across replicates and colored by treatment.
**Figure S3.** Accessions cluster by crop type, not differences in plant area. Reads were mapped to a concatenated reference of R500 and TO1000 and single‐nucleotide polymorphisms (SNPs) were called using GATK. Plant area was quantified as the number of pixels using the plantCV software. Pixels were averaged by treatment for each accession. Relative difference in plant area was calculated as the absolute value of water‐limited area minus well‐watered. PCAs were made using the ggplot2 package in RStudio 4.3.1.
**Figure S4.** *B. napus* subgenomes exhibit slight bias in transcript levels but treatment levels are the same. Transcript counts were normalized and the log sum was used to compare expression levels of all expressed genes by accession. Accessions are listed along the x‐axis.
**Figure S5.** Subgenome contribution varies by time of day and accession. Phase groups were calculated by subtracting the timing of peak expression (phase) of WL from the phase of WW for every gene and accession. Proportions were calculated as the number of genes by subgenome in a given phase group relative to the total number of genes for that accession/subgenome.
**Figure S6.** Some accessions have higher chlorophyll fluorescence under prolonged water limitation but have little differences in net photosynthetic efficiency or chlorophyll content. Fv/Fm reflectance (a) and chlorophyll fluorescence (b) images were captured using the CropReporter (PhenoVation Life Sciences, Wageningen, The Netherlands), and were analyzed using PlantCV v3.14.1. Joyplots were created using the “pcv.joyplot” function of the pcvr package (Sumner et al., 2023) in R v4.3.1 (R Core Team, 2020). Values were normalized for plant size and averaged across replicates. Histograms are colored by value. Vertical lines indicate peak (mode) of the well‐watered treatment on day 21. (c) Total chlorophyll content was measured from leaf tissue in well‐watered (WW) and water‐limited (WL) plants at ZT6. Accessions are grouped along the x‐axis. Ne and St had significantly higher (*P* = 0.038) and lower (*P* = 0.003) chlorophyll content after 3 weeks of water limitation.
**Figure S7.** Accessions do not show signs of stress under prolonged water limitation. Non‐photochemical quenching of chlorophyll fluorescence (NPQ; a) and normalized difference vegetation index (NDVI; b) reflectance images were captured using the CropReporter (PhenoVation Life Sciences, Wageningen, The Netherlands) and were analyzed using PlantCV v3.14.1. Joyplots were created using the “pcv.joyplot” function of the pcvr package (Sumner et al., 2023) in R v4.3.1 (R Core Team, 2020). Values were normalized for plant size and averaged across replicates. Histograms are colored by value. Higher NPQ values indicate more light energy being dissipated in the form of heat and therefore not being used by the plants for photosynthesis, while higher NDVI values indicate greener plants. Vertical lines indicate peak (mode) of the well‐watered treatment on day 21.
**Figure S8.** St and Mu maintain similar carbon assimilation rates throughout 4 weeks of water limitation. With a single exception (Mu; 24 days) there was no significant difference between treatments in carbon assimilation (A = μmols of assimilated carbon per square meter per second) indicating accessions had adjusted to the treatment. Measurement days are indicated at the bottom of each panel. Asterisks indicate a significant difference between treatments (*** is *P* = <0.001). Yellow boxes indicate water limited; green are well‐watered.
**Figure S9.** Variation in glucosinolate content in a spring and winter oil. Aliphatics (A) and a single aromatic (B) were quantified over an 18 hour time course for well‐watered (green) and water‐limited (yellow) plants. White (lights on) and black (lights off) show the relative time of day for each set of samples. Some aliphatics completely fell below the limit of detection (LOD; St: GJV; Mu: GJV; St: SIN) as indicated by a dashed red line and shaded box. Others were less consistent (St: NAP; PRO) as they were detectable but still below our limit of quantification (dashed black line) at some time points. NAP = Guconapin; PRO = Progoitrin; SIN = Sinigrin; GJV = Glucoputranjivin; NAS = Gluconasturin. Asterisks indicate a significant difference between treatments (*P ≤ 0.05; **P ≤ 0.01; ***P ≤ 0.001).
**Figure S10.** Prior predictive check for area growth model. We used lognormal priors for each estimated parameter in our growth model (A, B, C) and in the sub‐model for variance (subA, subB, subC). For the asymptote parameter we used a lognormal distribution with mu = 130 and sigma = 0.25. For the inflection point parameter we used a lognormal distribution with mu = 15 and sigma = 0.25. For the growth rate parameter we used a lognormal distribution with mu = 0.25 and sigma = 0.25. Prior predictive checks were conducted using the plotPrior function from the pcvr R package (Sumner et al., 2023).
**Figure S11.** Prior predictive check for increase in variance sub‐model. We used lognormal priors for each estimated parameter in our growth model (A, B, C) and in the sub‐model for variance (subA, subB, subC). For the asymptote parameter we used a lognormal distribution with mu = 130 and sigma = 0.25. For the inflection point parameter we used a lognormal distribution with mu = 15 and sigma = 0.25. For the growth rate parameter we used a lognormal distribution with mu = 0.25 and sigma = 0.25. Prior predictive checks were conducted using the plotPrior function from the pcvr R package (Sumner et al., 2023).

**Table S1.** Table of Gompertz growth model parameters and hypotheses.

**Table S2.** Table of the Kruskal–Wallis Chi‐squared test results for the NIR phenotyping data for all accessions.

**Table S3.** Metadata table for the transcriptome analysis. Contains the total number and list of genes identified with significantly different expression patterns or levels in response to water limitation (Metadata tab). Information on the crop types and origin of the 16 *Brassica napus* accessions used in the study (CropTypeInfo). GO analysis results for the DiPALM clusters and ResponseScore modules. List of enriched sulfur and GSL terms that were identified from the GO analysis.

**Table S4.** Table of the glucosinolates analyzed in the metabolomics analysis. Mass ranges used for extracting the glucosinolate features for St and Mu including the retention start and end times for detection.

## Data Availability

All raw and processed data have been made publicly available. Images from the phenotyping experiments are available on Cyverse (https://datacommons.cyverse.org/browse/iplant/home/shared/danforth_center/Ricono_et_al_Brassica). RNA sequencing data are available through the NCBI GEO database (GSE261928), reviewer token: yrizcwcitfghbwl. All scripts used to analyze the image data can be found on github (https://github.com/danforthcenter/ktgreenham‐brassica‐drought‐paper). All code used to analyze the RNA sequencing data including the scripts used to generate the figures can be found on github (https://github.com/GreenhamLab/Bnapus_WL_TranscriptomeTC). Raw metabolomics data can be found at the Data Repository for the University of Minnesota (DRUM; https://hdl.handle.net/11299/264077) by searching the manuscript title. Software used in this study is open‐source or available for free.
