# Supplementary figures and images for: Homoeolog expression divergence contributes to time of day changes in transcriptomic and glucosinolate responses to prolonged water limitation in Brassica napus
Source: Plant J. 2025 Feb 24;121(4):e70011. doi: 10.1111/tpj.70011 (PMC11849911; doi:10.1111/tpj.70011)

Figure S1

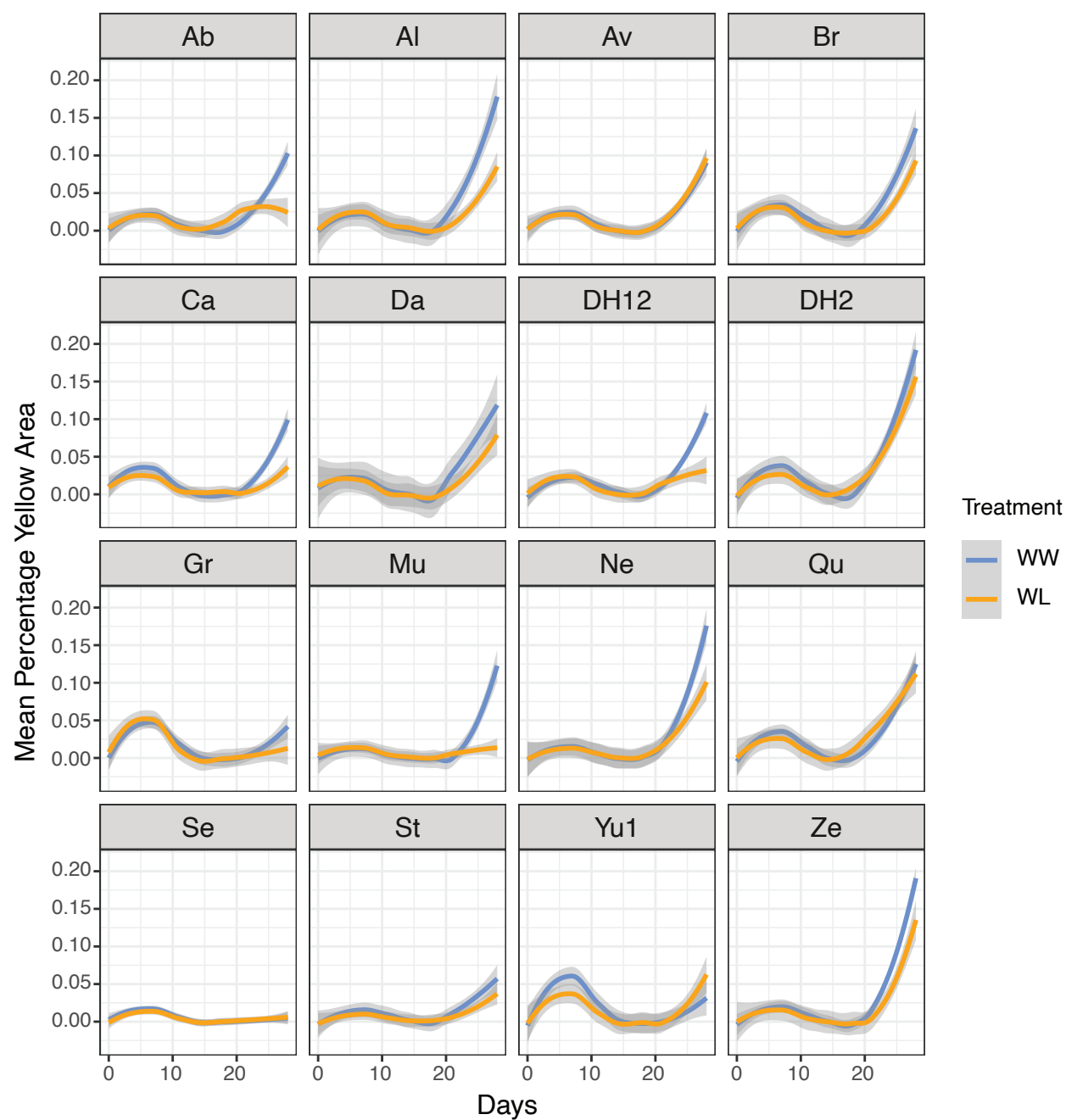

Figure S2

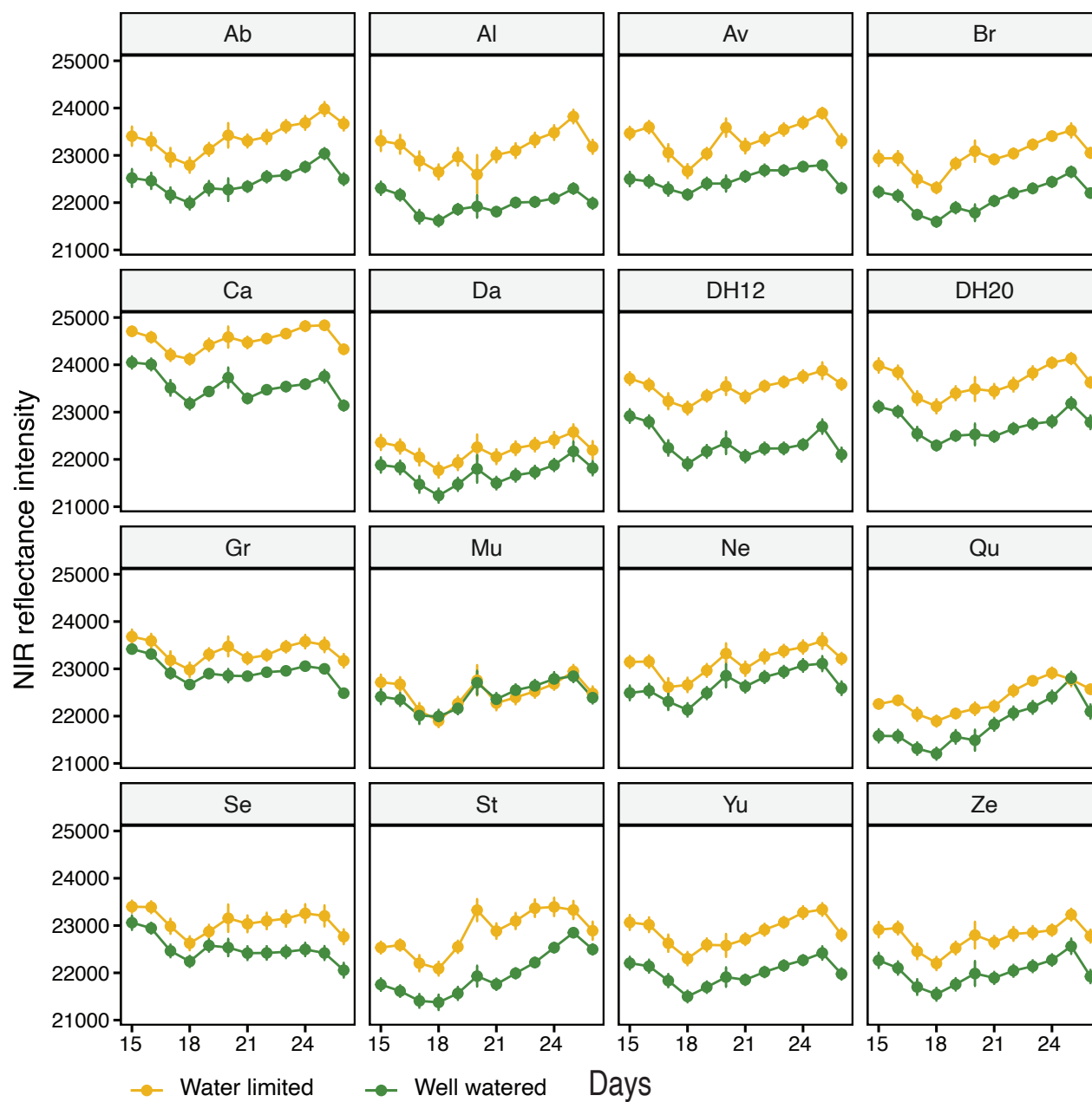

Figure S3

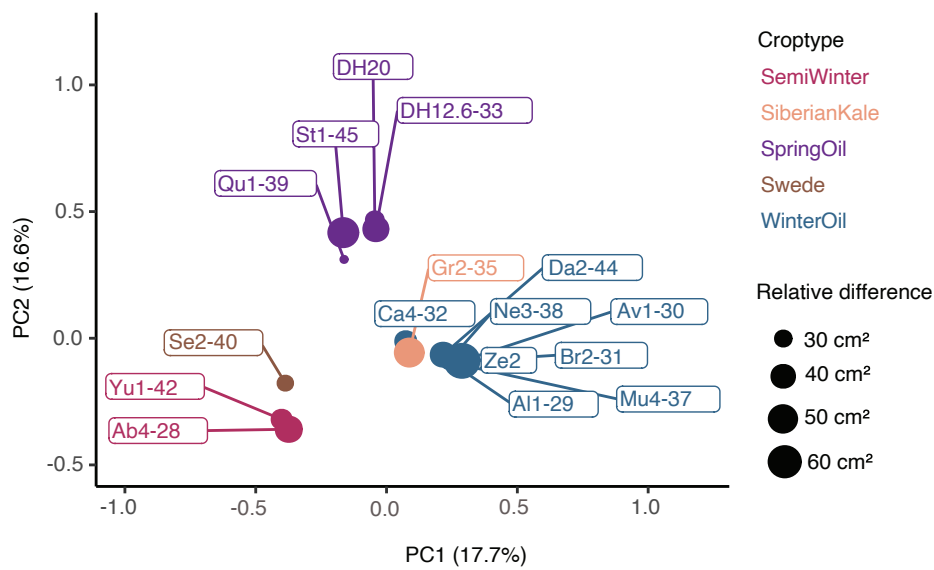

Figure S4

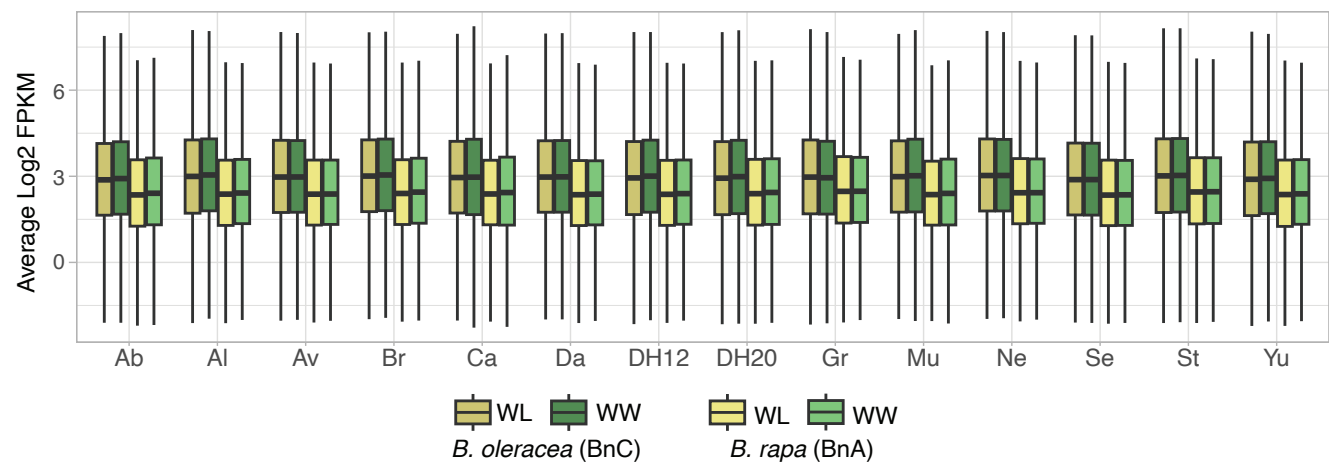

Figure S5

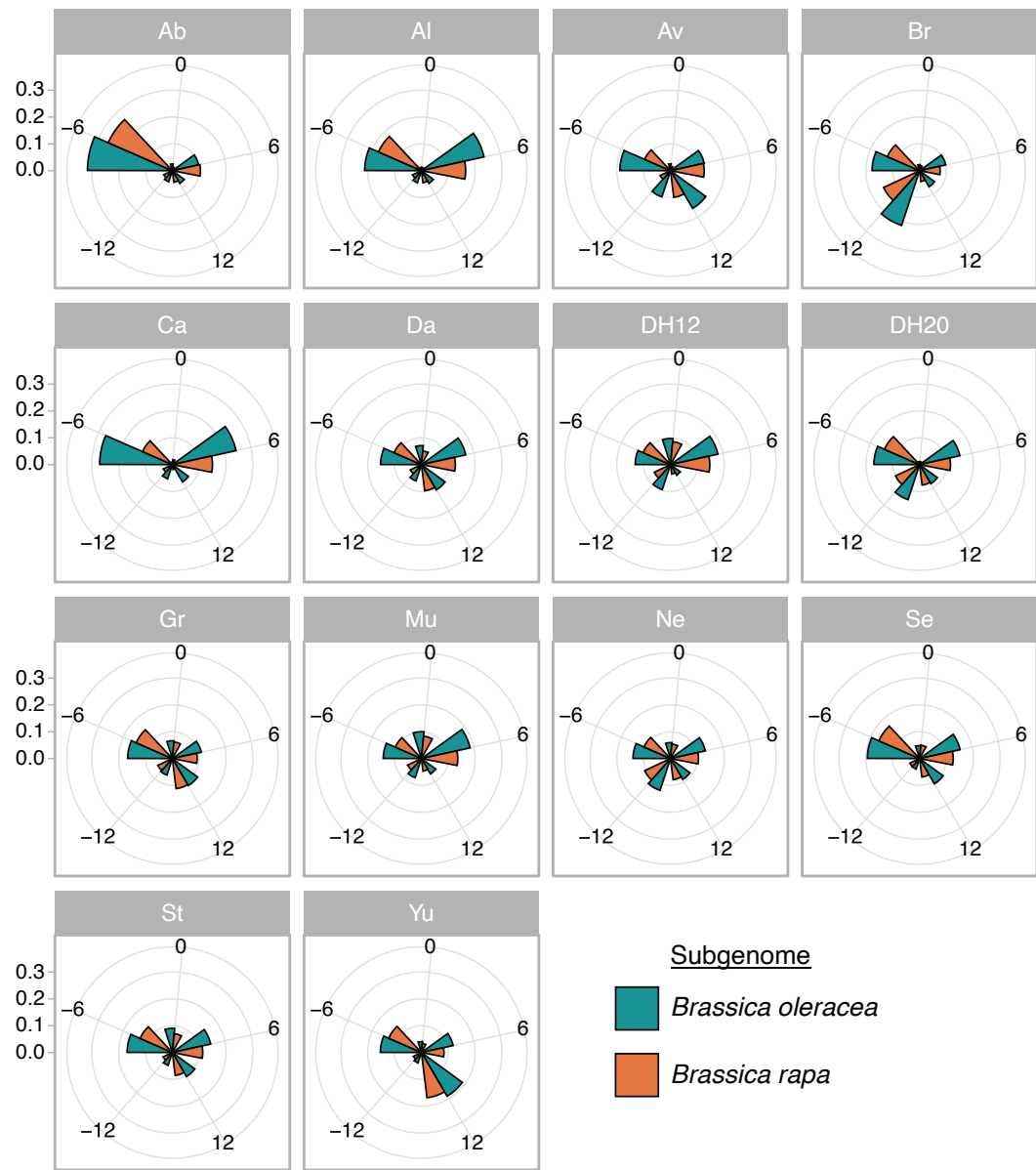

Figure S6

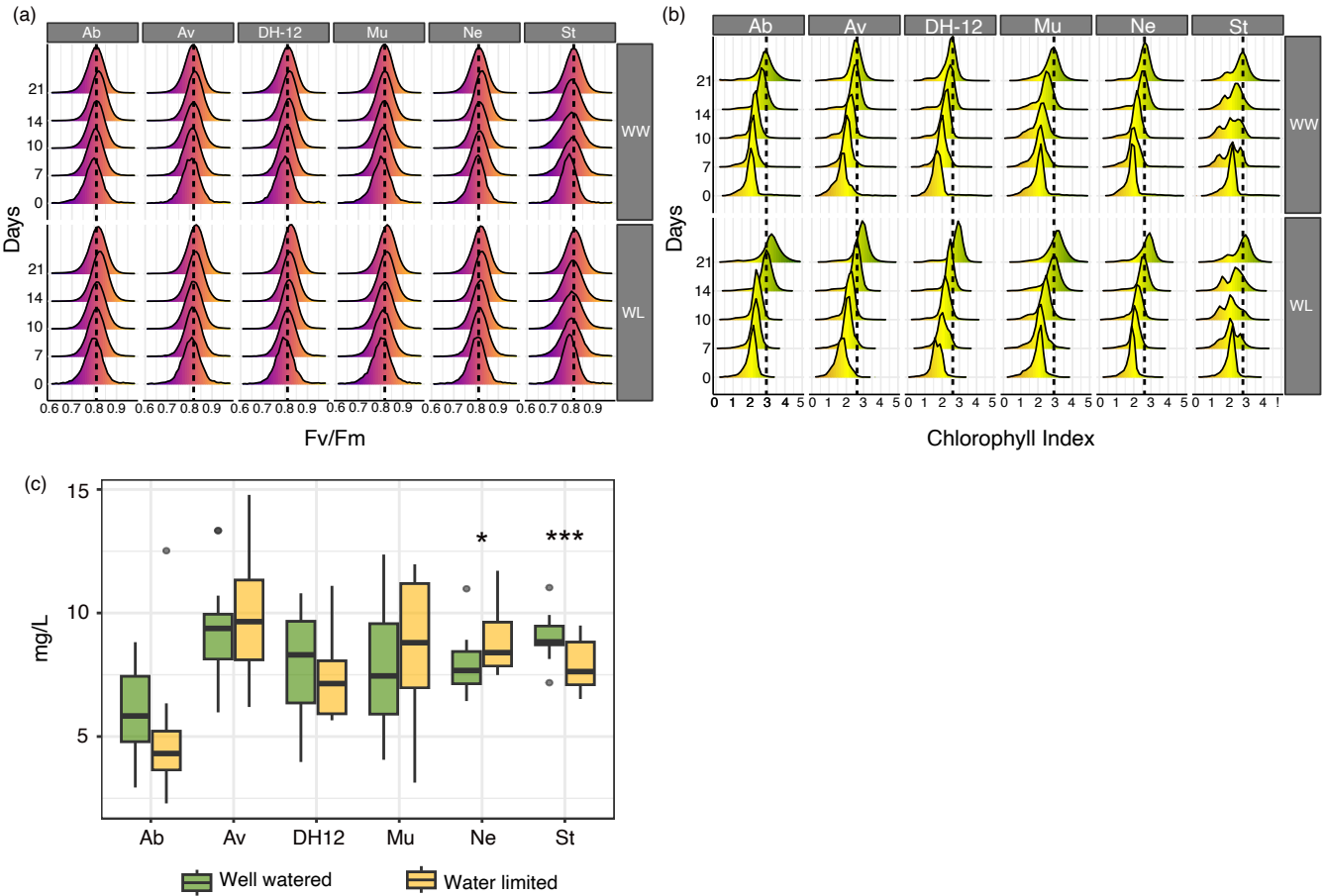

Figure S7

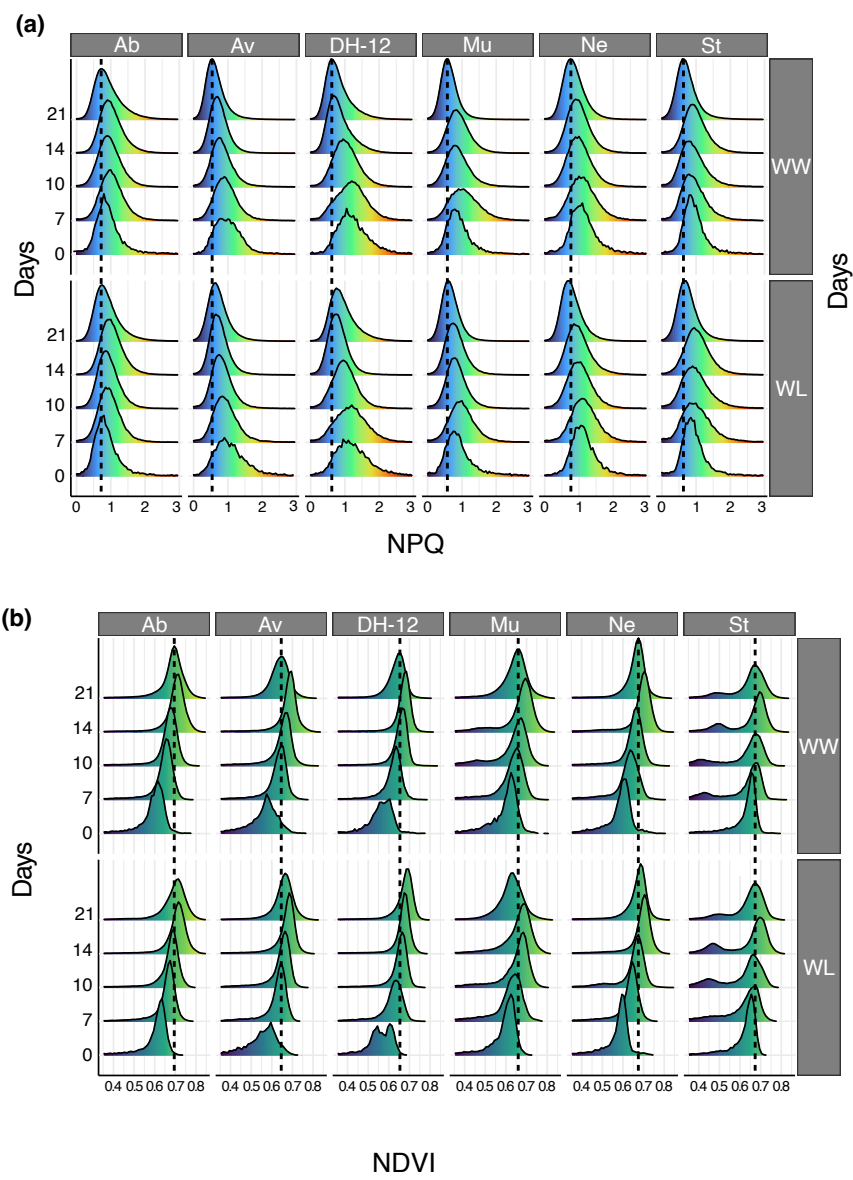

Figure S8

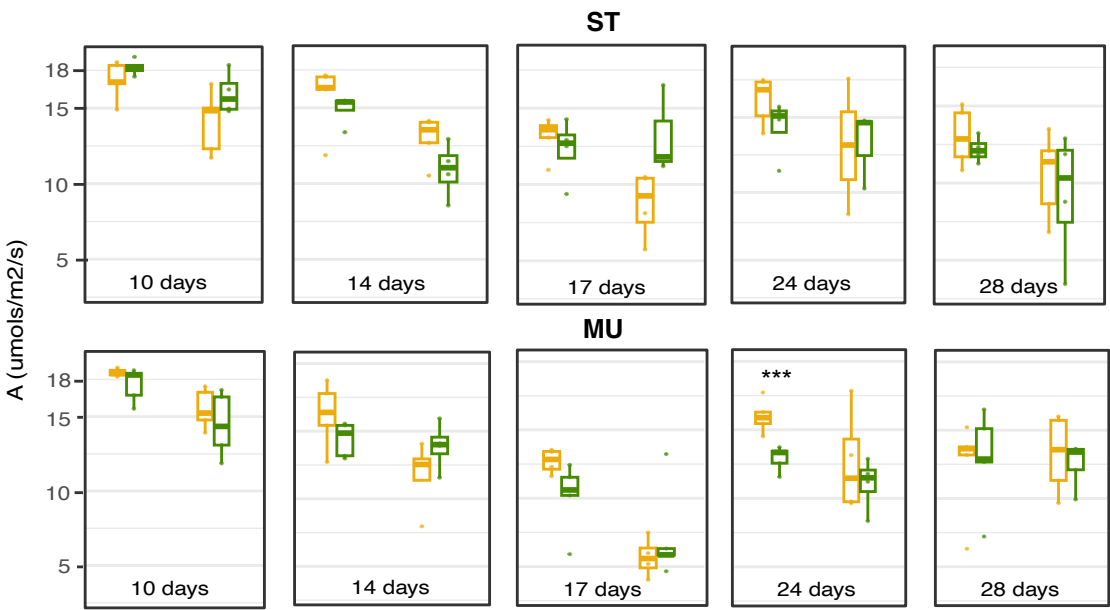

Figure S9

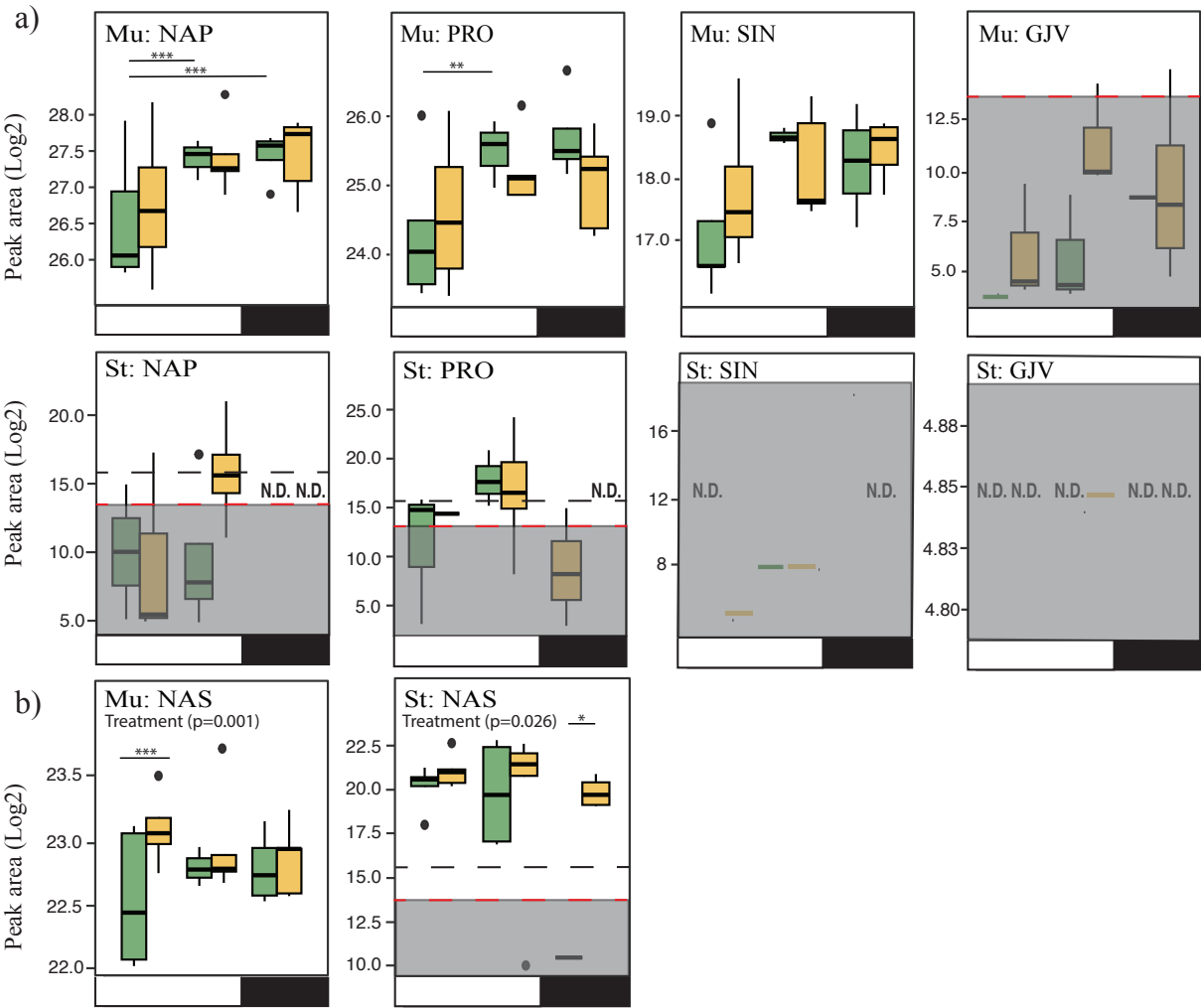

Figure S10

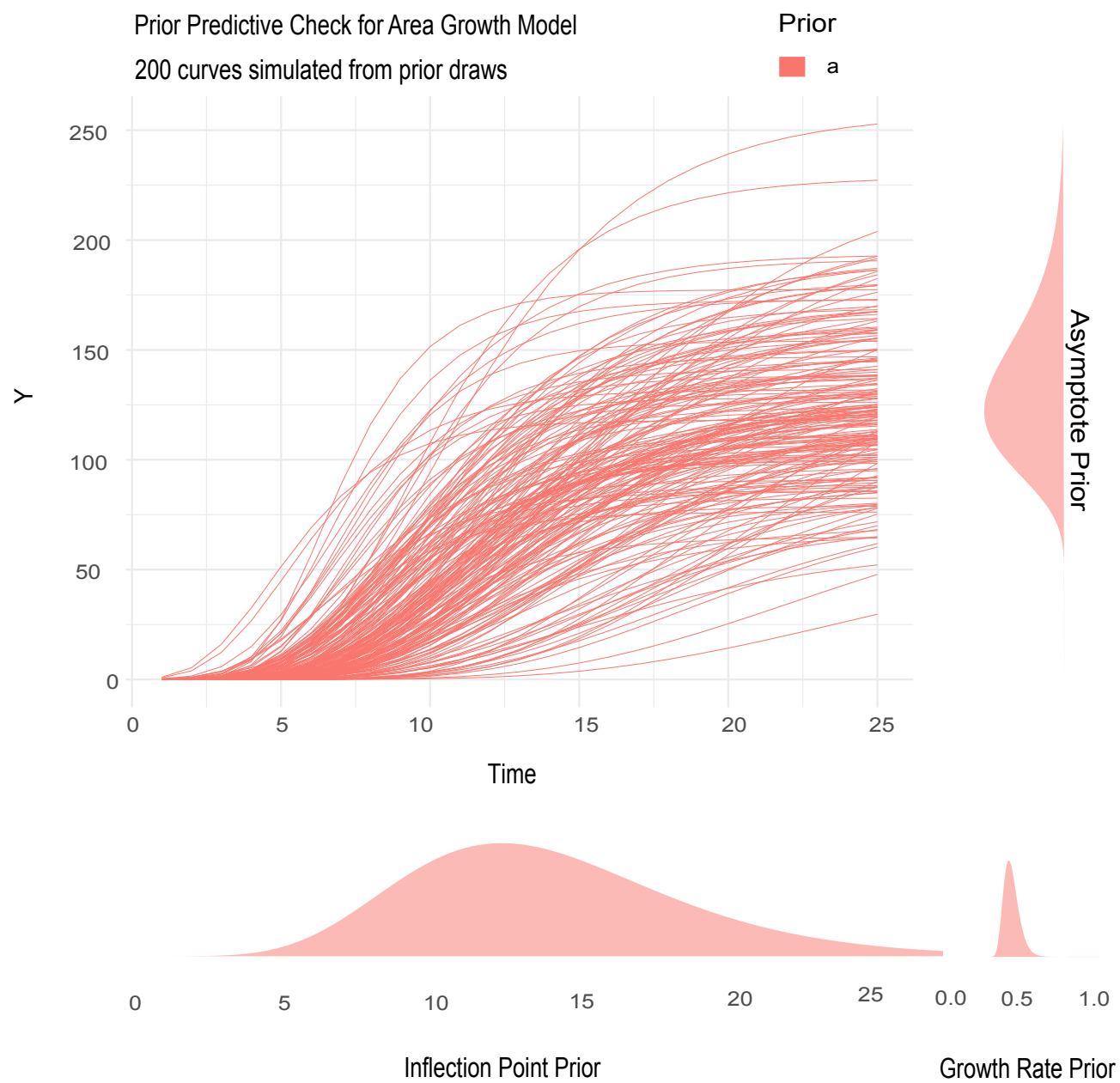

Figure S11

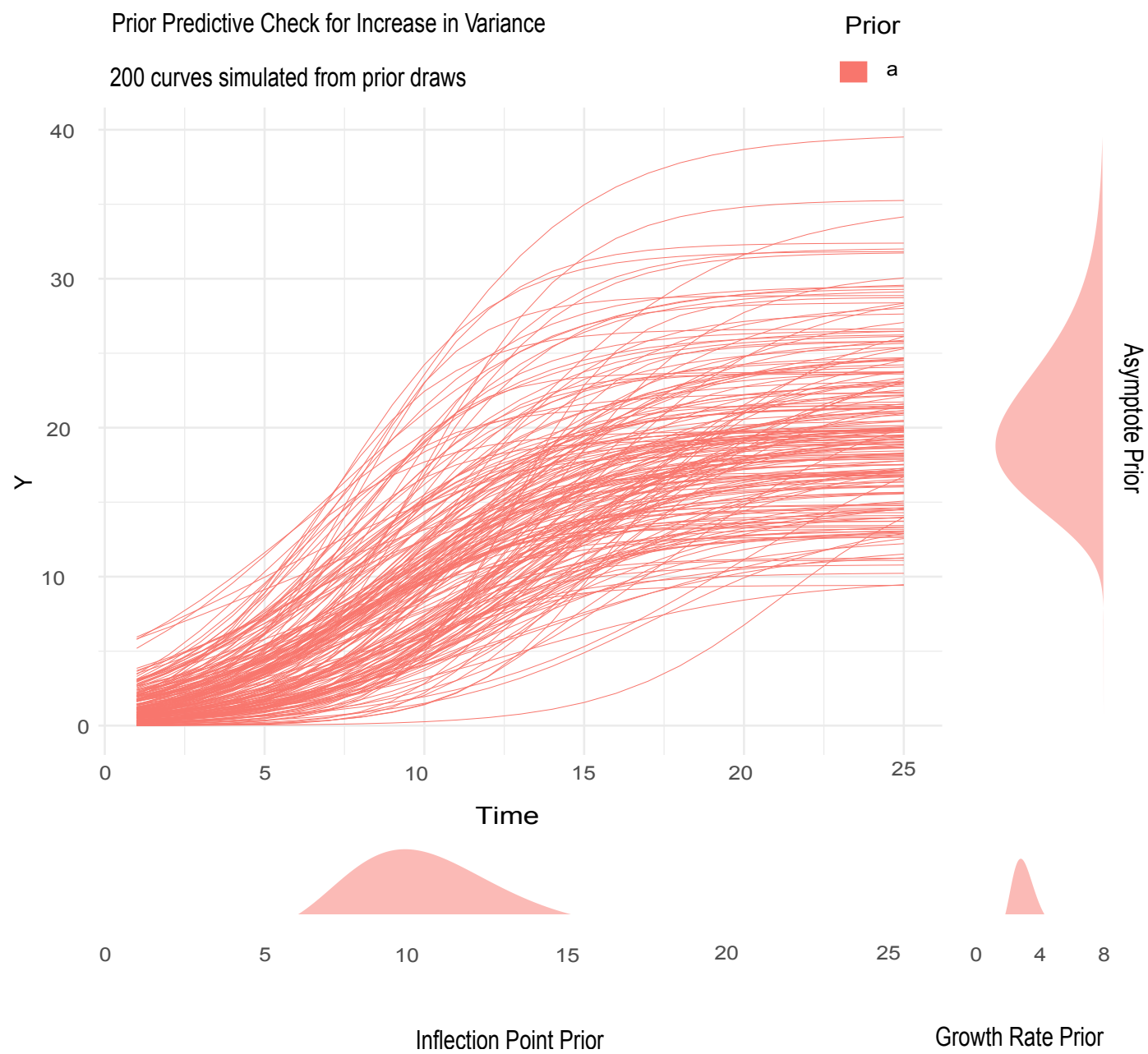

Supplement: Supplementary file 1 — Figure S1. Percent of plant pixels classified as “yellow” by a Naive Bayes classifier. Brassica napus accessions exhibit reduced growth with water limitation (Figure 1). Several accessions on average have a lower percentage of plant pixels classified as ‘yellow’ at the end of the water limitation treatment compared with the well‐watered treatment. Figure S2. NIR signal is higher in most plants in water‐limited treatment, suggesting lower water content than in well‐watered plants. Only NIR reflectance from day 14 to day 26 are shown, when images were taken at a consistent zoom level. Values were averaged across replicates and colored by treatment. Figure S3. Accessions cluster by crop type, not differences in plant area. Reads were mapped to a concatenated reference of R500 and TO1000 and single‐nucleotide polymorphisms (SNPs) were called using GATK. Plant area was quantified as the number of pixels using the plantCV software. Pixels were averaged by treatment for each accession. Relative difference in plant area was calculated as the absolute value of water‐limited area minus well‐watered. PCAs were made using the ggplot2 package in RStudio 4.3.1. Figure S4. B. napus subgenomes exhibit slight bias in transcript levels but treatment levels are the same. Transcript counts were normalized and the log sum was used to compare expression levels of all expressed genes by accession. Accessions are listed along the x‐axis. Figure S5. Subgenome contribution varies by time of day and accession. Phase groups were calculated by subtracting the timing of peak expression (phase) of WL from the phase of WW for every gene and accession. Proportions were calculated as the number of genes by subgenome in a given phase group relative to the total number of genes for that accession/subgenome. Figure S6. Some accessions have higher chlorophyll fluorescence under prolonged water limitation but have little differences in net photosynthetic efficiency or chlorophyll content. Fv/Fm reflectan [file TPJ-121-0-s003.pdf]
